# Human AGR2 Deficiency Causes Mucus Barrier Dysfunction and Infantile Inflammatory Bowel Disease

**DOI:** 10.1016/j.jcmgh.2021.07.001

**Published:** 2021-07-06

**Authors:** Ahmad A. Al-Shaibi, Ussama M. Abdel-Motal, Satanay Z. Hubrack, Alex N. Bullock, Amna A. Al-Marri, Nourhen Agrebi, Abdulrahman A. Al-Subaiey, Nazira A. Ibrahim, Adrian K. Charles, COLORS in IBD-Qatar Study Group, Saad R. Al-Kaabi, Muneera J. Al-Mohannadi, Fayaz A. Mir, Hamad Medical Corporation, Doha, Qatar, Holm H. Uhlig, Simon P.L. Travis, University of Oxford, Oxford, UK, Mamoun Elawad, Anthony K. Akobeng, Nazira A. Ibrahim, Fatma Al-Mudahka, Bernice Lo, Sidra Medicine, Doha, Qatar, Mamoun Elawad, Holm H. Uhlig, Bernice Lo

**Affiliations:** 1 Research Branch, Sidra Medicine, Doha, Qatar; 4 Department of Gastroenterology, Sidra Medicine, Doha, Qatar; 5 Department of Anatomical Pathology, Sidra Medicine, Doha, Qatar; 2 Centre for Medicines Discovery, University of Oxford, Oxford, United Kingdom; 6 Translational Gastroenterology Unit, John Radcliffe Hospital, University of Oxford, Oxford, United Kingdom; 3 College of Health and Life Sciences, Hamad Bin Khalifa University, Doha, Qatar; 7 Oxford Biomedical Research Centre, Oxford, United Kingdom; 8 Department of Pediatrics, University of Oxford, Oxford, United Kingdom

**Keywords:** AGR2, MUC2, ER Stress, Intestinal Metaplasia, Goblet Cells, AGR2, anterior gradient 2, BiP, Binding Immunoglobulin Protein, CLCA1, Chloride Channel Accessory 1, ER, endoplasmic reticulum, HA, hemagglutinin, HEK293T, Human Embryonic Kidney 293T, IBD, inflammatory bowel disease, MUC, mucin, TBS, Tris-buffered saline, TFF3, trefoil factor 3, WT, wild-type

## Abstract

**Background & Aims:**

The gastrointestinal epithelium plays a crucial role in maintaining homeostasis with the gut microbiome. Mucins are essential for intestinal barrier function and serve as a scaffold for antimicrobial factors. Mucin 2 (MUC2) is the major intestinal gel-forming mucin produced predominantly by goblet cells. Goblet cells express anterior gradient 2 (AGR2), a protein disulfide isomerase that is crucial for proper processing of gel-forming mucins. Here, we investigated 2 siblings who presented with severe infantile-onset inflammatory bowel disease.

**Methods:**

We performed whole-genome sequencing to identify candidate variants. We quantified goblet cell numbers using H&E histology and investigated the expression of gel-forming mucins, stress markers, and goblet cell markers using immunohistochemistry. AGR2-MUC2 binding was evaluated using co-immunoprecipitation. Endoplasmic reticulum (ER) stress regulatory function of mutant AGR2 was examined by expression studies in Human Embryonic Kidney 293T (HEK293T) using tunicamycin to induce ER stress.

**Results:**

Both affected siblings were homozygous for a missense variant in *AGR2*. Patient biopsy specimens showed reduced goblet cells; depletion of MUC2, MUC5AC, and MUC6; up-regulation of AGR2; and increased ER stress. The mutant AGR2 showed reduced capacity to bind MUC2 and alleviate tunicamycin-induced ER stress.

**Conclusions:**

Phenotype–genotype segregation, functional experiments, and the striking similarity of the human phenotype to *AGR2*^*-/-*^ mouse models suggest that the AGR2 missense variant is pathogenic. The Mendelian deficiency of AGR2, termed “Enteropathy caused by AGR2 deficiency, Goblet cell Loss, and ER Stress” (EAGLES), results in a mucus barrier defect, the inability to mitigate ER stress, and causes infantile-onset inflammatory bowel disease.

## Graphical abstract

**Figure:**
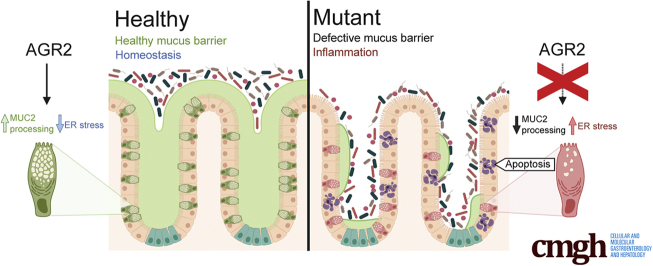

**Summary**

This report describes the discovery of a human anterior gradient 2 deficiency causing monogenic infantile inflammatory bowel disease resulting from goblet cell depletion and a mucus barrier defect.

Extreme early onset of inflammatory bowel disease (IBD) in neonates (neonatal IBD) enabled the discovery of many monogenic forms of IBD. Disease variants affecting immunity and epithelial barrier function have been described,^1^, ^2^, ^3^ which show the crucial role each play in gastrointestinal mucosal homeostasis.

Creating a physical barrier between the intestinal lumen and the body is a key function of the epithelial layer. Among the many mechanisms used by the gastrointestinal epithelium to block bacterial access, the formation of a mucus barrier is paramount.^4^ In the intestines, the highly glycosylated, gel-forming mucin, mucin 2 (MUC2), is produced by goblet cells and forms the multilayered matrix of the mucus barrier. Deficiency of *Muc2* in mice disrupts the barrier and results in gastrointestinal inflammation.^5^^,^^6^ Defective Muc2 glycosylation in mouse models also impairs the mucus barrier, enabling direct contact between bacteria and the intestinal epithelial surface.^7^ Another key function of mucus in the gastrointestinal tract is to protect epithelial cells from environmental insults, such as gastric acid.^8^^,^^9^

MUC2 processing involves a multitude of proteins, among which is anterior gradient 2 (AGR2), an endoplasmic reticulum (ER) protein and a member of the protein disulfide isomerase family^10^ that aids in the folding of proteins in the ER. Within the gut, *AGR2* is expressed predominantly in goblet cells.^11^ Genetic variants in the 5’ or promoter region of *AGR2* have been found to be associated with IBD in cohorts of German and UK patients.^12^ AGR2 regulates ER stress^13^ and plays an important role in the processing and production of the gel-forming mucins Mucin 2,^14^^,^^15^ Mucin 5AC, and Mucin 5B.^16^^,^^17^ ER stress itself is a process contributing to IBD in humans.^12^^,^^18^^,^^19^ Studies in mouse models have confirmed a pertinent role for ER stress in IBD and have shown that intestinal secretory cells (ie, goblet cells and Paneth cells) are especially susceptible to ER stress. For instance, knockout of the ER stress signaling protein X-Box Binding Protein (Xbp1) in mouse intestinal epithelial cells leads to ER stress and the apoptotic loss of goblet cells and Paneth cells, resulting in the spontaneous development of intestinal inflammation.^20^ Misfolding of MUC2 alone also can trigger goblet cell ER stress. In the *Winnie* and *Eeyore* mouse models, Muc2 contains missense mutations that prevent it from being processed properly. These mice present with increased ER stress of goblet cells, resulting in the loss of goblet cells, an impaired mucus barrier, and the spontaneous development of colitis.^21^ These studies highlight the sensitivity of goblet cells to ER stress and the importance of proper MUC2 processing in maintaining goblet cell health and overall mucosal homeostasis.

Agr2 is crucial for mucus production and mucosal homeostasis as shown in mouse studies. *Agr2* knockout mice show loss of intestinal mucus, increased susceptibility to dextran sodium sulfate–induced colitis,^15^ and develop spontaneous ileitis and colitis accompanied by markers of increased ER stress.^14^ Goblet cells of these mice show increased accumulation of non–O-glycosylated MUC2,^22^ indicating a defect in MUC2 processing as a consequence of AGR2 loss.

In this study, we investigated siblings diagnosed with congenital diarrhea who developed severe infantile IBD. Both patients showed a striking absence of goblet cells by histology. The patients were homozygous for a missense loss-of-function variant in AGR2. Our work identifies a novel Mendelian epithelial defect that causes intestinal inflammation as a consequence of a defective mucous barrier.

## Results

### Clinical Presentation

We studied 2 male siblings (patient 1 and patient 2) who presented with congenital diarrhea and infantile-onset IBD born to a consanguineous couple. Both were born at term and had a normal weight at delivery. Both patients presented with diarrhea since birth and poor weight gain (<0.01 percentile). There were no other family members with similar symptoms. At 6 weeks of age, the diarrhea of patient 1 worsened upon the introduction of cow milk–based formula. Patient 1 was admitted to the hospital at 3 months of age with failure to thrive and was switched to an amino acid–based formula, which resulted in a slight improvement of his diarrhea. Based on the medical history of his brother, patient 2 was kept on exclusive breast feeding before switching to an amino acid–based formula very early, which helped to improve his symptoms to some extent. An extensive immunologic work-up (Tables 1 and 2) for both patients showed no indication of a primary immune deficiency (such as autoimmune enteropathy, lymphocyte differentiation defects, chronic granulomatous disease, or immunoglobulin deficiency). A detailed clinical history is included in the Supplementary Materials section.

**Table 1:** Immune Profile of Patient 1

Cell type	Value	Reference range
Complete blood count, *×10*^*9*^*/L*^a^		
Neutrophil	10.4	0.8–7.2
Lymphocyte	4.4	1.3–8
Monocytes	1	0.1–10
Eosinophils	0.1	0–0.7
Basophils	0	0–0.2
Total immunoglobulins, *g/L*		
Test		
IgG	14.96	4.68–13.28
IgA	3.79	0.44–1.87
IgM	0.75	0.31–1.51
Lymphocyte subsets, *cells/mcL*^a^		
Markers		
CD3	3426	900–4500
CD3+/CD4+	1775	500–2400
CD3+/CD8+	1434	300–1600
CD19	471	200–2100
CD3-/CD16/CD56+	586	100–1000

NOTE. All tests were performed at 4 years of age.

a Absolute values.

**Table 2:** Immune Profile of Patient 2

Cell type	Value	Reference range
Complete blood count, *×10*^*9*^*/L*^a^		
Neutrophil	3.3	0.97–5.45
Lymphocyte	7.31	2.45–8.89
Monocytes	1.29	0.28–1.07
Eosinophils	1.01	0.03–0.61
Basophils	0.14	0.01–0.06
Total immunoglobulins, *g/L*		
Test		
IgG	821	172–814
IgA	66.9	8.1–84
IgM	254	33–108
Lymphocyte subsets, *cells/mcL*^a^		
Markers		
CD3	4114	2300–6500
CD3+/CD4+	2274	1500–5000
CD3+/CD8+	1756	500–1600
CD19	1812	600–3000
CD3-/CD16/CD56+	941	100–1300

NOTE. All tests were performed at 4 months of age.

a Absolute values.

### Infantile Enteropathy With Intestinal Goblet Cell Loss and Intestinal Metaplasia of the Gastric Epithelium

**Figure 1:**
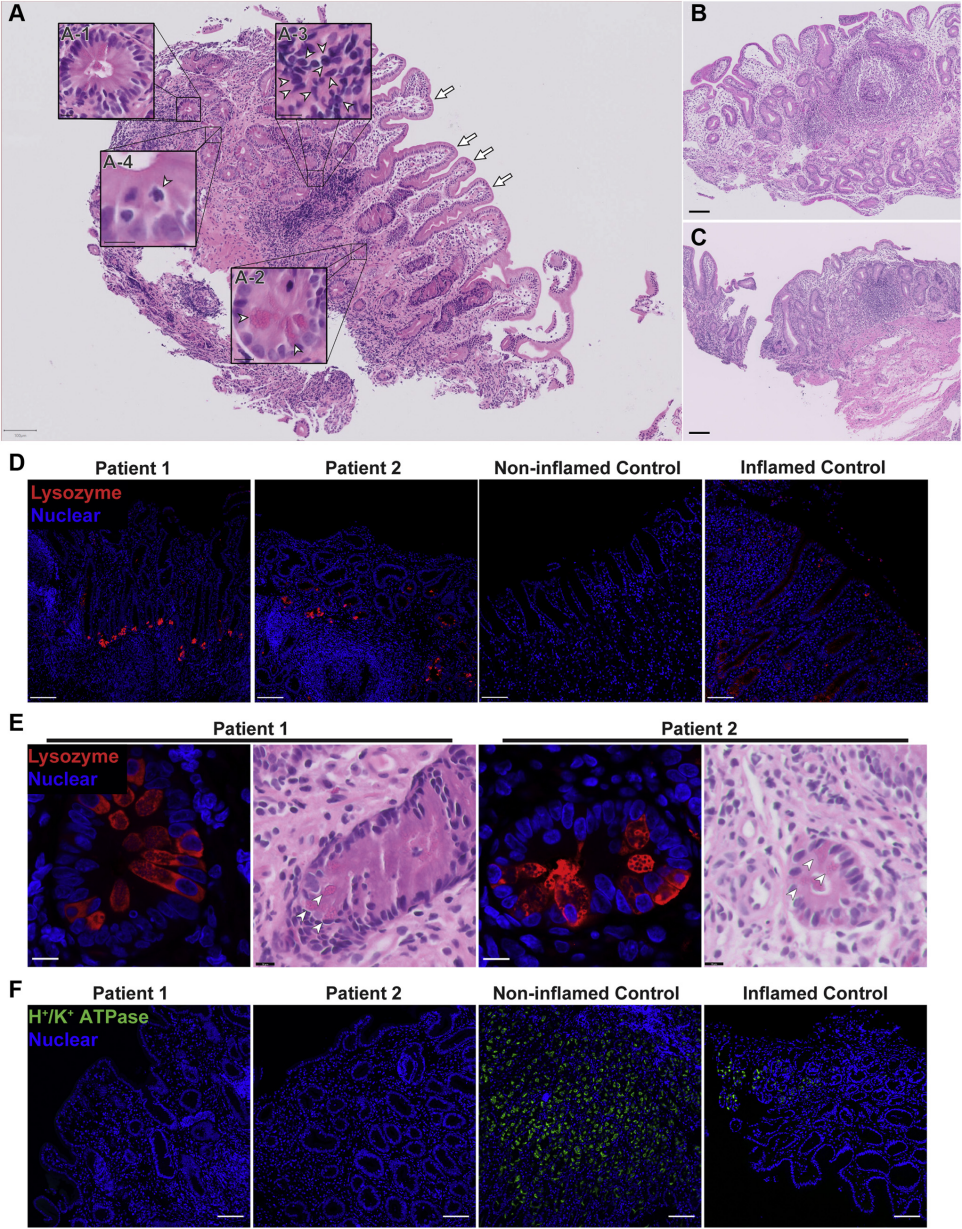
**Patients developed severe intestinal metaplasia in the gastric epithelium with the appearance of Paneth cells and loss of parietal cells.** (*A–C*) Scans of H&E staining on formalin-fixed paraffin-embedded (FFPE) gastric sections were used to highlight metaplastic and inflammatory features of the patients. *Insets*: *Arrowheads* indicate the feature described in the legend pertaining to that particular inset. (*A*) Gastric section of patient 1 taken at 1 year of age showing severe intestinal metaplasia and patchy lymphocytic infiltration. Metaplasia was shown by the presence of villiforms (*arrows*), crypt-like structures (*A-1*), granulated Paneth-like cells (*A-2*), and a lack of recognizable parietal and foveolar cells. Inflammation is evident by the presence of patchy lymphocytic infiltrates (*A-3*), and mitotic figures indicate a regenerative state (*A-4*). Scans from gastric section of (*B*) patient 2 taken at 2 months of age alongside (*C*) patient 1 at 6 months of age showing the same extensive metaplasia and metaplastic features. Biopsies were performed at (*A* and *B*) Hamad General Hospital, and (*C*) Al Wakra Hospital. Each biopsy was performed by a different physician. (*A–C*) H&E slides were acquired with a slide scanner using a 40× objective. *Insets* were generated using digital zoom. (*D*) Lysozyme was detected by fluorescent immunohistochemistry (IHC) and confocal microscopy on FFPE sections. Nuclei were stained with Hoechst (blue). Lysozyme (red, polyclonal antibody) expression in the stomachs of patients was increased and localization was concentrated in the Paneth-like cells. (*A–D*) *Scale bars*: 100 μm. (*E*) Higher magnification of Paneth-like cells in the gastric epithelium via H&E and fluorescent IHC shows these cells expressed lysozyme (red) and were highly granular and eosinophilic. Thus, these gastric Paneth-like cells highly resemble the Paneth cells of the small bowel. *Small panels* and *insets*: *Scale bars*: 10 μm. (*F*) Fluorescent immunohistochemistry of parietal cell marker H^+^/K^+^ adenosine triphosphatase (ATPase) (green, clone C-4) performed on FFPE sections. Nuclei were stained with Hoechst (blue). The staining shows depletion of H^+^/K^+^ ATPase, which is consistent with the loss of parietal cells on H&E. H^+^/K^+^ ATPase staining was replicated 3 independent times with 2 inflamed controls and 2 noninflamed controls. Inflamed control shown in the figure is positive for *H pylori*. Lysozyme staining was replicated in at least 3 independent experiments including a total of 3 noninflamed controls and 4 inflamed controls. Identical thresholding parameters for each marker were used.

Histologically, gastric sections showed patchy lymphocytic infiltration (Figure 1*A*). In addition, the gastric mucosa showed extensive intestinal metaplasia manifesting with the appearance of villiforms, crypts, an absence of recognizable parietal cells, and the presence of eosinophilic Paneth-like cells (Figure 1*A*, *D*, and *E*). Scans of biopsy specimens from both patients taken at the ages of 6 months and 1 year for patient 1, and at age 2 months for patient 2 are shown in Figure 1*A–C*. All biopsy specimens showed similar, extensive intestinal metaplasia with prominent Paneth-like cells and an absence of gastric morphology. The observed Paneth-like cells in the gastric epithelium were highly granular and expressed lysozyme, which is a typical Paneth cell marker (Figure 1*D* and *E*). Foveolar cells, which are responsible for the bulk of gastric surface mucus production, also were not detectable on H&E sections owing to the loss of their characteristic thecae (Figure 1*A*). Immunohistochemical staining for the parietal cell marker H^+^/K^+^ adenosine triphosphatase (H^+^/K^+^ATPase) showed that both patients were negative, indicating an absence of parietal cells (Figure 1*F*).

**Figure 2:**
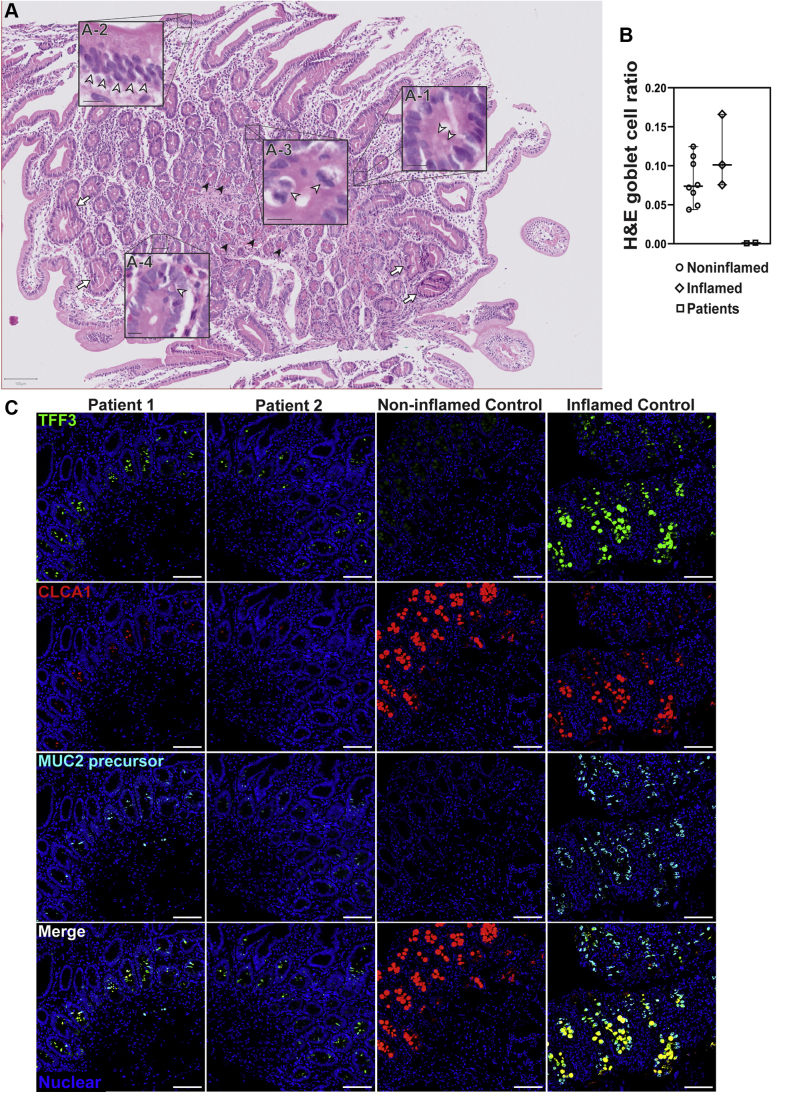
**Patients 1 and 2 show severe loss of goblet cells in the small intestines.** (*A*) Patient small bowel was nearly devoid of goblet cells on H&E staining of formalin-fixed paraffin-embedded (FFPE) sections, with few potential examples of apical staining clearances consistent with the morphology and localization of goblet cell thecae (*A-1*). The section also shows disorganization of columnar epithelial cells (*A-2*). Mitotic figures (*A-3*) and regenerative crypts (*arrows*) indicate a regenerative state. Apoptotic bodies also are present (*A-4*), indicating ongoing apoptotic activity. Paneth cells appeared unremarkable (*black arrowheads*). *White arrowheads* point to the features highlighted in each respective inset. Images were acquired using a slide scanner with a 40× objective and were cropped digitally. Scale bars: 100 μm (main panel); 10 μm (magnified *insets*). Digital zoom was used for magnified *insets*. Representative section from patient 1 is shown here. Both patients showed identical presentation. (*B*) Quantification of goblet cell depletion in small-bowel H&E sections. Results are the ratio of goblet cells to total epithelial cells. Each *point* represents the data from 1 individual. *Line* represents the median and *bars* indicate 95% CI. (*C*) Fluorescent immunohistochemistry staining for the goblet cell markers TFF3 (green, clone B-1), CLCA1 (red, clone EPR12254-88), and nonglycosylated MUC2 precursor (turquoise, clone CCP58) in paraffin-embedded sections from small bowel detected by a laser scanning microscope using a 10× objective. Nuclei were stained with Hoechst (blue). Signal was acquired from the entire thickness of the section by adjusting the pinhole to cover 9 μm. Each marker was replicated at least 3 independent times. CLCA1 was visualized using a goat anti-rabbit AF647 antibody and goat anti-mouse AF546 for TFF3. Nonglycosylated MUC2 antibody was conjugated directly with AF594. For small bowel, TFF3 staining was replicated across 4 noninflamed controls and 5 inflamed controls, while CLCA1 and nonglycosylated MUC2 precursor staining were replicated across 2 noninflamed controls and 2 inflamed controls. Small-bowel inflamed control shown in the figure is from a patient diagnosed with Familial Mediterranean fever.

**Figure 3:**
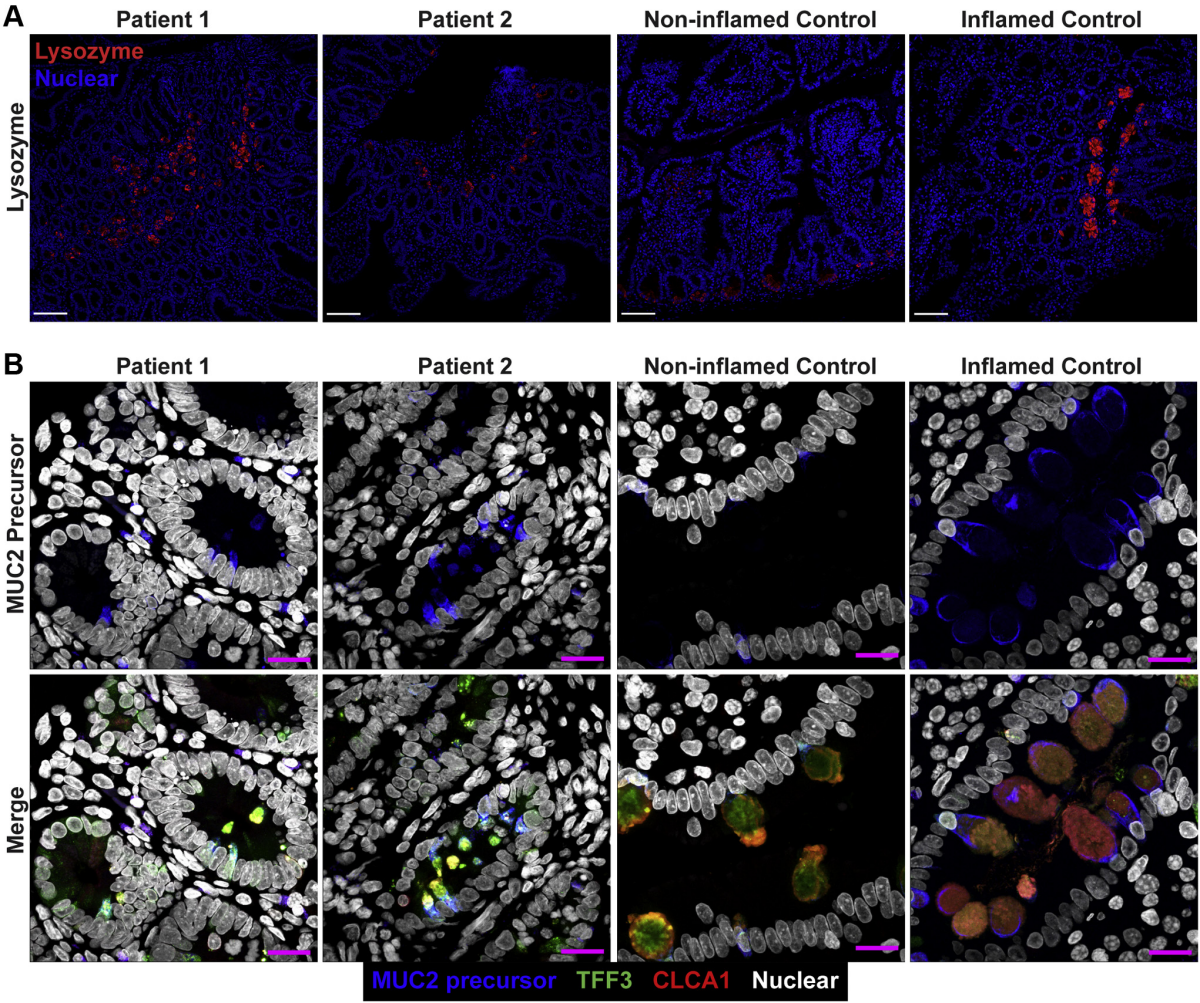
**Small bowel of the patients show unremarkable localization of lysozyme in Paneth cells and depletion of nonglycosylated MUC2 precursor compared with inflamed controls.** (*A*) Confocal images of fluorescent immunohistochemical staining for lysozyme (red, polyclonal) shows normal localization of Paneth cells in the small intestines of both siblings. Confocal acquisition was performed using a 10× objective. *Scale bars*: 100 μm. Lysozyme staining was replicated in a minimum of 3 independent experiments including a total of 3 noninflamed controls and 4 inflamed controls. (*B*) Maximum intensity projection of confocal Z-stacks acquired from small-bowel sections stained for MUC2 precursor (blue, CCP58), TFF3 (green, B-1), and CLCA1 (red, EPR12254-88) with Hoechst nuclear counterstain (white). Staining shows little accumulation of MUC2 precursor in the patients compared with inflamed controls, but not with noninflamed controls, which appeared to have the lowest accumulation of the precursor. Patient sections also show reduced size of goblet cell thecae. Images were acquired using a 63× objective. *Scale bar*: 10 μm. Replicates included 2 noninflamed controls and 4 inflamed controls. Identical thresholding parameters for each marker were applied to all images in each respective panel.

Duodenal sections showed little to no cellular signs of inflammation, but a near-complete absence of goblet cells with regenerative crypts, mitotic figures, and apoptotic bodies (Figure 2*A*). To quantify the loss of goblet cells in the siblings observed in the H&E-stained sections, we calculated the ratio of goblet cells–to–total epithelial cells in H&E histology sections. In the small bowel, we compared patient sections (n = 2) with noninflamed (n = 8) and unrelated IBD sections (n = 3). In the small bowel of the siblings there was an extreme reduction in the percentage of goblet cells among epithelial cells (0.1%) compared with noninflamed (8.1%) and IBD (11.4%) controls (Figure 2*B*). To further evaluate the loss of goblet cells, we stained for the goblet cell markers trefoil factor 3 (TFF3/Intestinal Trefoil Factor), Chloride Channel Accessory 1 (CLCA1) and the nonglycosylated MUC2 precursor, using multiplexed immunohistochemistry. TFF3 and CLCA1 levels were found to be reduced dramatically in the siblings (Figure 2*C*) compared with controls. However, cells positive for TFF3, the nonglycosylated MUC2 precursor, and CLCA1 still were detectable in the crypts of the small-bowel mucosa of patients 1 and 2. Unlike the stomach, duodenal Paneth cells appeared unremarkable (Figure 3*A*). The nonglycosylated MUC2 precursor was reduced in the goblet cells of both patients compared with inflamed controls (Figure 3*B*).

**Figure 4:**
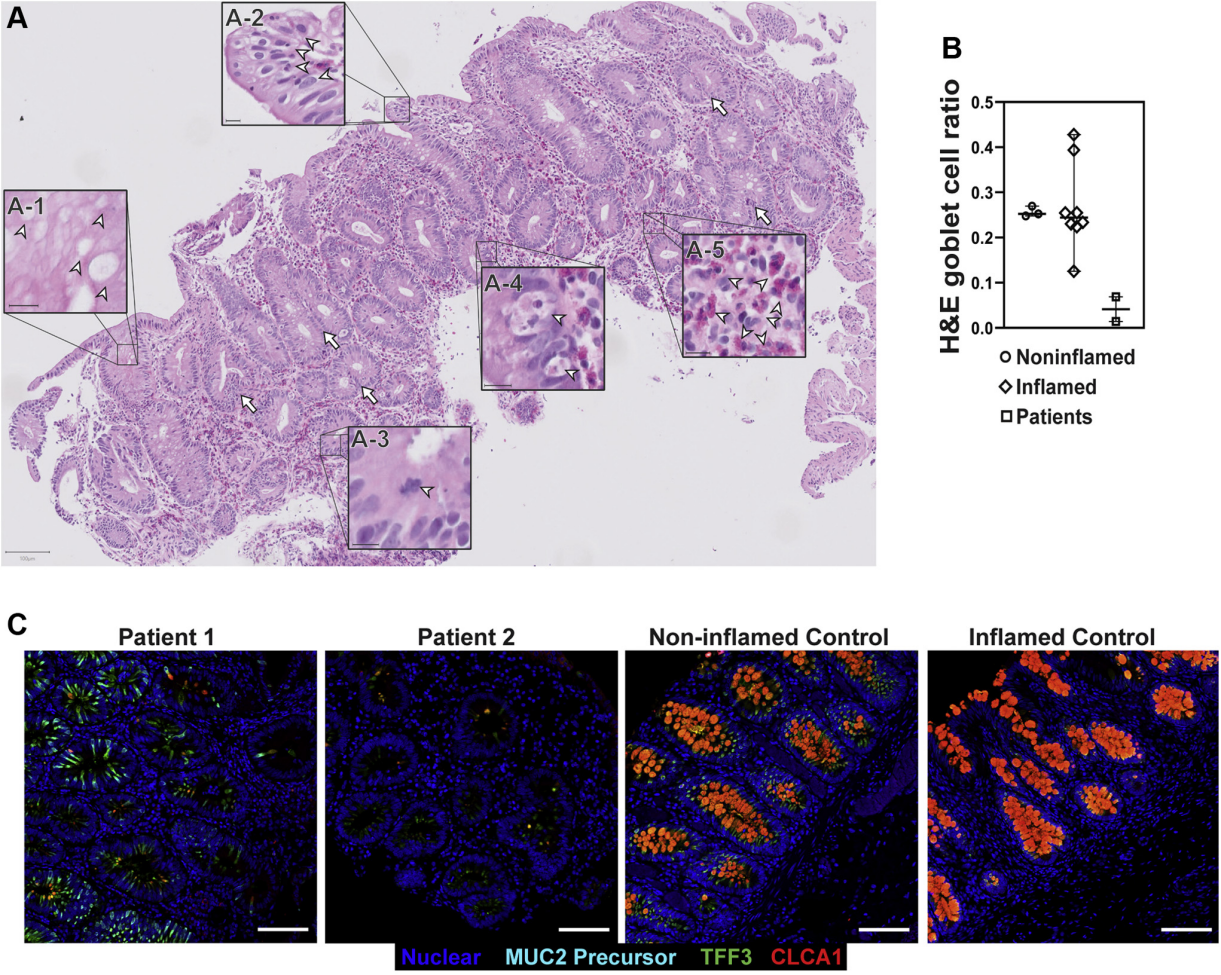
**Patient large bowel shows intense inflammation and loss of goblet cells.** (*A*) H&E scan of patient 1 large bowel shows reduction of goblet cells with some remaining clusters (*A-1*). Other features include epithelial disorganization (*A-2*), frequent mitotic (*A-3*) and apoptotic bodies (*A-4*), eosinophilic infiltration (*A-5*), and regenerative crypts (*arrows*). *Arrowheads* indicate the features of each respective inset. Scale bars: 100 μm (main panel); 10 μm (magnified *insets*). Digital zoom was used for magnified *insets*. (*B*) Quantification of goblet cell depletion in H&E sections of large bowel. Each *dot* represents the ratio of goblet cells to total epithelial cells from 1 individual. *Horizontal line* represents the median and *bars* indicate 95% CI. (*C*) Fluorescent immunohistochemistry staining for the goblet cell markers TFF3 (green, clone B-1), CLCA1 (red, clone EPR12254-88), and nonglycosylated MUC2 precursor (turquoise, clone CCP58) in paraffin-embedded sections from large bowel with nuclear counterstain (blue, Hoechst). Representative images are shown for patients 1 and 2. Each marker was replicated at least 3 independent times. CLCA1 was visualized using a goat anti-rabbit AF647 antibody and goat anti-mouse AF546 for TFF3. Nonglycosylated MUC2 antibody was conjugated directly with AF594. Images were acquired using a laser scanning confocal microscope with a 10× objective. The pinhole was adjusted to 9 μm to acquire signal from the entire thickness of the section. *Scale bar*: 100 μm. TFF3 staining was replicated across 3 noninflamed controls and 6 inflamed controls, while CLCA1 and nonglycosylated MUC2 precursor staining were replicated across 2 noninflamed controls and 3 inflamed controls. Large-bowel inflamed control shown in the figure is from a patient with appendicitis.

**Figure 5:**
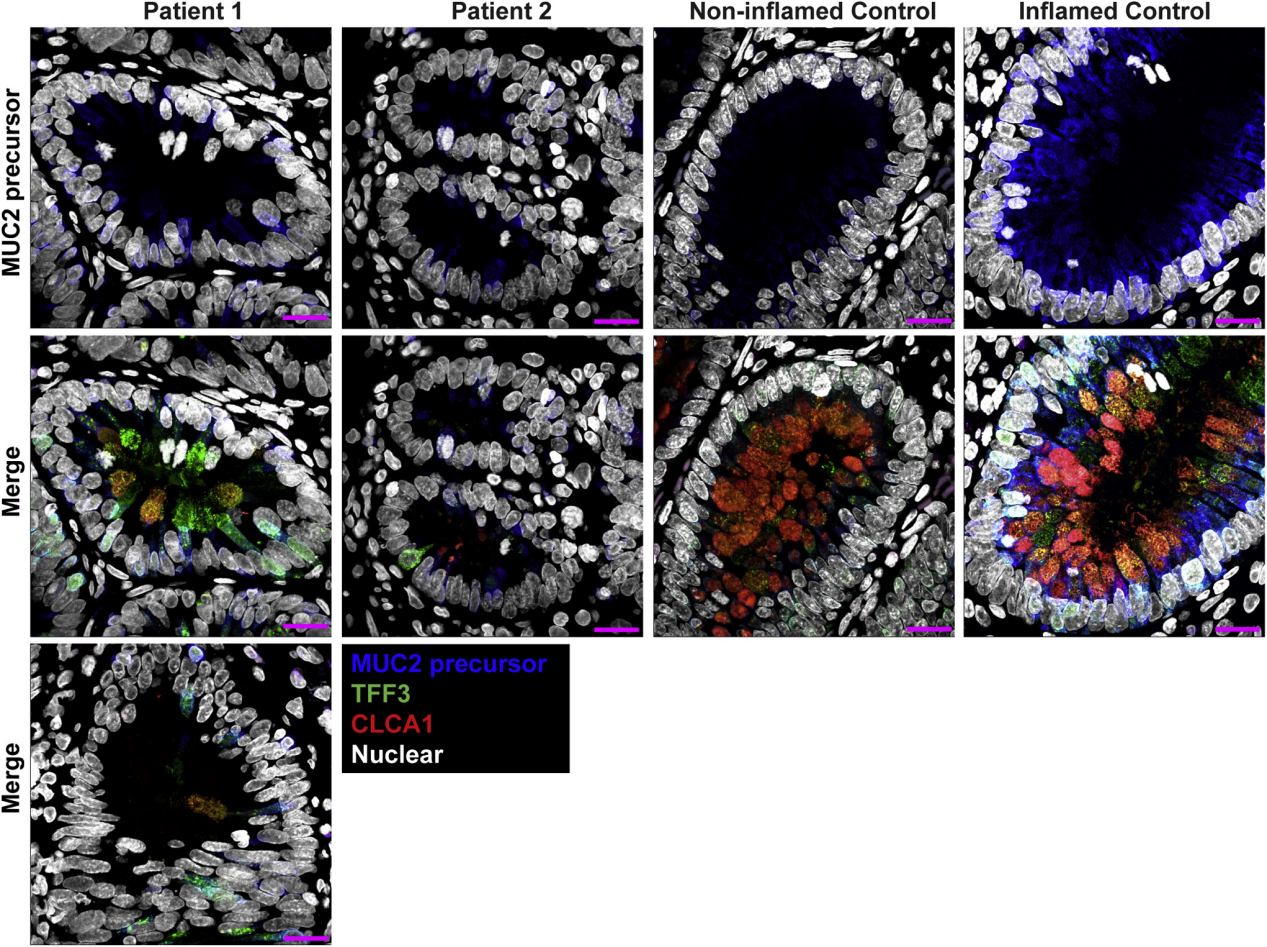
**Reduced accumulation of nonglycosylated MUC2 precursor within goblet cells of the large bowels of the patients compared with inflamed controls.** Maximum intensity projection of confocal Z-stacks acquired from large-bowel sections stained for MUC2 precursor (blue, CCP58), TFF3 (green, B-1), CLCA1 (red, EPR12254-88), and nuclei counterstained with Hoechst (white). Accumulation of MUC2 precursor appears reduced in the goblet cells of patients compared with other inflamed controls, but not compared with noninflamed controls, which appear to have the least amount of MUC2 precursor. Goblet cells of the patients also show reduced size of their thecae. For patient 1, 2 images from the large bowel are shown to illustrate the heterogeneity in goblet cell phenotype between crypts of the large bowel in this patient. Staining for all markers was repeated at least 3 independent times. *Scale bars*: 10 μm. Acquisition was performed with a laser scanning confocal microscope using a 63× objective. Staining was replicated across 2 noninflamed and 3 inflamed controls. Identical thresholding parameters for each marker were applied to all images.

Large-bowel sections showed the presence of plasma cells, conspicuous eosinophilic infiltration, apoptotic bodies, mitotic figures, and regenerative crypts, all alongside a severe depletion of goblet cells (Figure 4*A*). Quantification of goblet cells in the colon in H&E-stained sections was performed using the same method for the small bowel. Patient sections (n = 2) were compared with noninflamed (n = 3) and unrelated IBD (n = 8) controls and showed a severe decrease in goblet-to-epithelial cell ratio (4.1%) compared with noninflamed (25.7%) and IBD (26.8%) controls (Figure 4*B*). Using fluorescent staining of TFF3, CLCA1, and nonglycosylated MUC2 precursor, we observed clusters of goblet cells in large-bowel crypts of patients 1 and 2. Goblet cell abundance still was reduced in the large bowels of patients compared with controls. The reduction appeared most significant in the small bowel but varied in the large bowel between patients. Patient 2 showed more severe depletion of goblet cells in his large bowel compared with patient 1 (Figure 4*C*). The thecae of these remaining goblet cells appeared to be reduced dramatically in size compared with noninflamed and inflamed controls, and accumulation of nonglycosylated MUC2 precursor in the large-bowel goblet cells of the patients also was reduced compared with inflamed controls (Figure 5).

The initial working diagnosis was of an autoimmune enteropathy as a result of the colitis, lack of goblet cells, and increased apoptosis.

### Whole-Genome Sequencing Identifies a Biallelic AGR2 Defect

**Figure 6:**
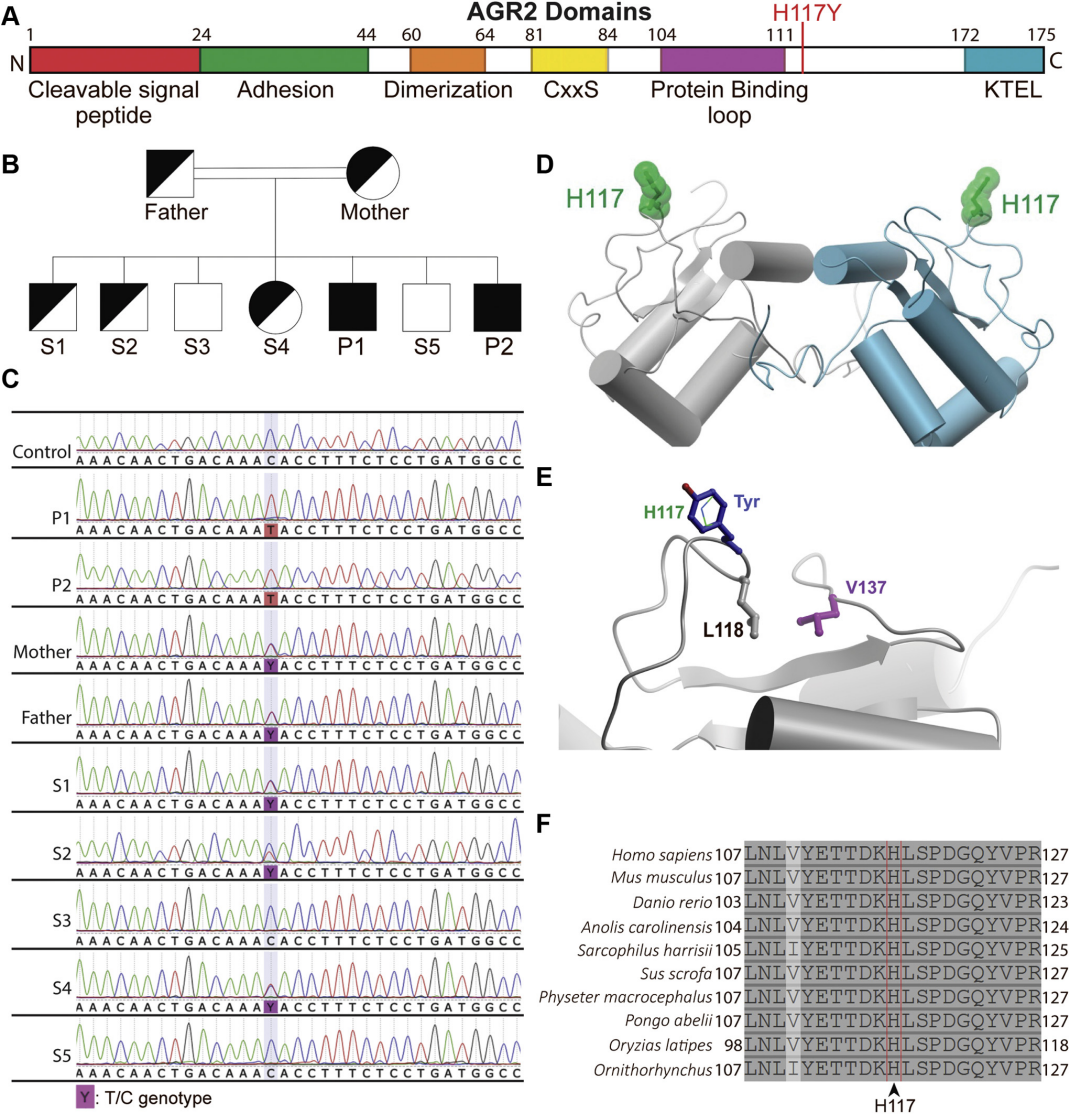
**Identification of a homozygous missense variant in *AGR2*.** (*A*) Map of AGR2 protein domains as reviewed by Delom et al^24^ with the mutation site indicated. (*B*) Pedigree showing the parents, the patients (P1, P2), and healthy siblings (S1–S5). Only the patients are homozygous for the mutation. (*C*) Chromatograms from Sanger sequencing of genomic DNA from the family and an unrelated healthy control. (*D*) Ribbon diagram of the WT AGR2 homodimer (grey/cyan, Protein Data Bank ID: 2LNS) showing the side chain position of H117 (green) at the protein surface in each monomer. (*E*) Surface location of H117 (green, thin stick representation) and modeling of the H117Y mutant side chain (blue) using the ICM software package (Molsoft). The neighboring residue L118 (grey) packs against V137 (purple, distance 4.3 Å) as shown. (*F*) H117 residue of AGR2 protein is conserved across species. Multiple sequence alignment of AGR2 orthologues, using Clustal Omega (UCD Conway Institute, Dublin, Ireland), and similarity highlighting were performed through UniProt. The following orthologues are shown with the UniProt identifiers in parentheses in the following order: humans (O95994), mouse (O88312), zebrafish (Q5RZ65), American chameleon (G1KMJ8), Tasmanian devil (G3VGV8), pig (A0A4X1TD21), sperm whale (A0A2Y9FEZ9), Sumatran orangutan (Q5R7P1), Japanese rice fish (A0A3B3IHX1), and Duckbill platypus (F7FAI5).

Whole-genome sequencing of the affected siblings and the parents showed both siblings to be homozygous for a variant in AGR2: c.349C>T; p.H117Y (rs780638101). Sanger sequencing of the parents, affected siblings, and healthy siblings confirmed autosomal-recessive inheritance of the variant (Figure 6*A–C*). Both parents and 3 of the 5 healthy siblings were heterozygous for the AGR2 variant. The variant is not present in the 1000 Genomes Project and only 1 heterozygote was found (allele frequency, 4 × 10^-6^) in the Genome Aggregation Database (gnomAD), indicating that the variant is extremely rare. No homozygous essential loss-of-function variants (ie, stop codon or frameshift) in *AGR2* were present in public genome databases. The variant leads to the substitution of histidine 117 by tyrosine in the AGR2 protein (Figure 6*D* and *E*). In silico analysis by various pathogenicity prediction scores SIFT (Craig Venter Institute, La Jolla, CA) (0.009), PolyPhen-2 (Harvard University, Boston, MA) (0.947), and Combined Annotation Dependent Depletion^23^ (CADD, https://cadd.gs.washington.edu/snv) (27.0) predicted the mutation to be deleterious. Other homozygous coding variants shared between both patients were considered unlikely to be pathogenic (Table 3). Analysis of the known 3-dimensional structure of AGR2 showed that the H117 side chain occupies a surface-exposed position that is not predicted to alter the overall protein structure (Figure 6*D*). However, this mutation is likely to impact AGR2 protein interactions because the mutation resides close to a predicted protein binding interface.^24^ Of note, H117 is adjacent to L118, which packs against V137, a residue that, when mutated in mice, impaired mucus production and resulted in intestinal inflammation and goblet cell loss similar to *Agr2* knockout mice^25^ (Figure 6*E*). Furthermore, the amino acid H117 is evolutionarily conserved across species (Figure 6*F*).

**Table 3:** Exonic Homozygous Variants Shared Between Patients

Gene	Variant	PolyPhen	SIFT	MutationTaster
*AGR2*	rs780638101	Possibly damaging	Damaging	Disease causing
*MAGEB3*	rs763926751	Benign	Tolerated	Polymorphism
*CXorf67*	rs781890238	Benign	Tolerated	Polymorphism
*HS6ST2*	rs1343137006	Benign	Damaging	Polymorphism
*CD99L2*	rs782738455	Benign	Tolerated	Polymorphism
*MAGEA4*	rs572691118	Benign	Tolerated	Polymorphism
*SLC6A8*	rs868950793	Benign	Damaging	Disease causing

The familial segregation, the near absence of the variant in population databases, the evolutionary conservation, and the absence of alternative pathogenic variants suggested p.H117Y to be a plausible candidate disease-causative variant.

### Altered AGR2 Protein Expression and Localization in Patients

**Figure 7:**
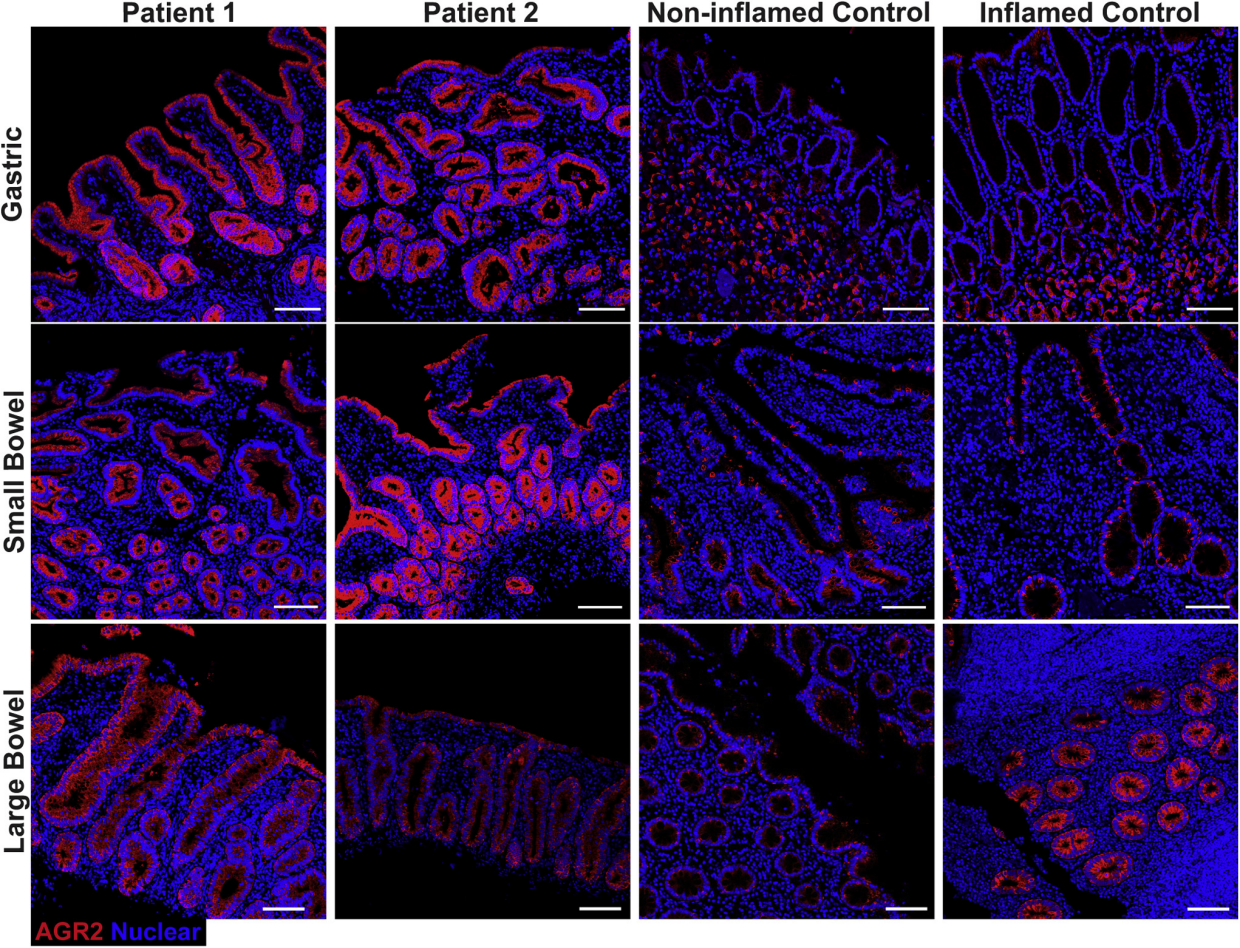
**Up-regulation and altered localization of AGR2 in gastrointestinal tissues of the patients.** Confocal images showing AGR2 (red, clone 5G1.1 for gastric and D9V2F for bowel) immunofluorescence staining on small and large intestinal and gastric sections. Intestinal stains were visualized with goat anti-rabbit AF647 secondary while gastric stains were visualized with goat anti-mouse AF546 secondary. Nuclei were stained with Hoechst (blue). Representative images are shown for patients 1 and 2. For small bowel, representative images are shown for 1 of 3 noninflamed controls and 1 of 5 inflamed controls. For large bowel, representative images are shown for 1 of 3 noninflamed controls and 1 of 6 inflamed controls. For gastric sections, representative images are shown for 1 of 3 noninflamed controls and 1 of 4 inflamed controls. Staining was replicated independently at least 3 times. Inflamed controls shown in the figure included ulcerative colitis for gastric, Crohn’s for small bowel, and appendicitis for large bowel. *Scale bar*: 100 μm. Identical thresholding parameters were applied for each row of images.

**Figure 8:**
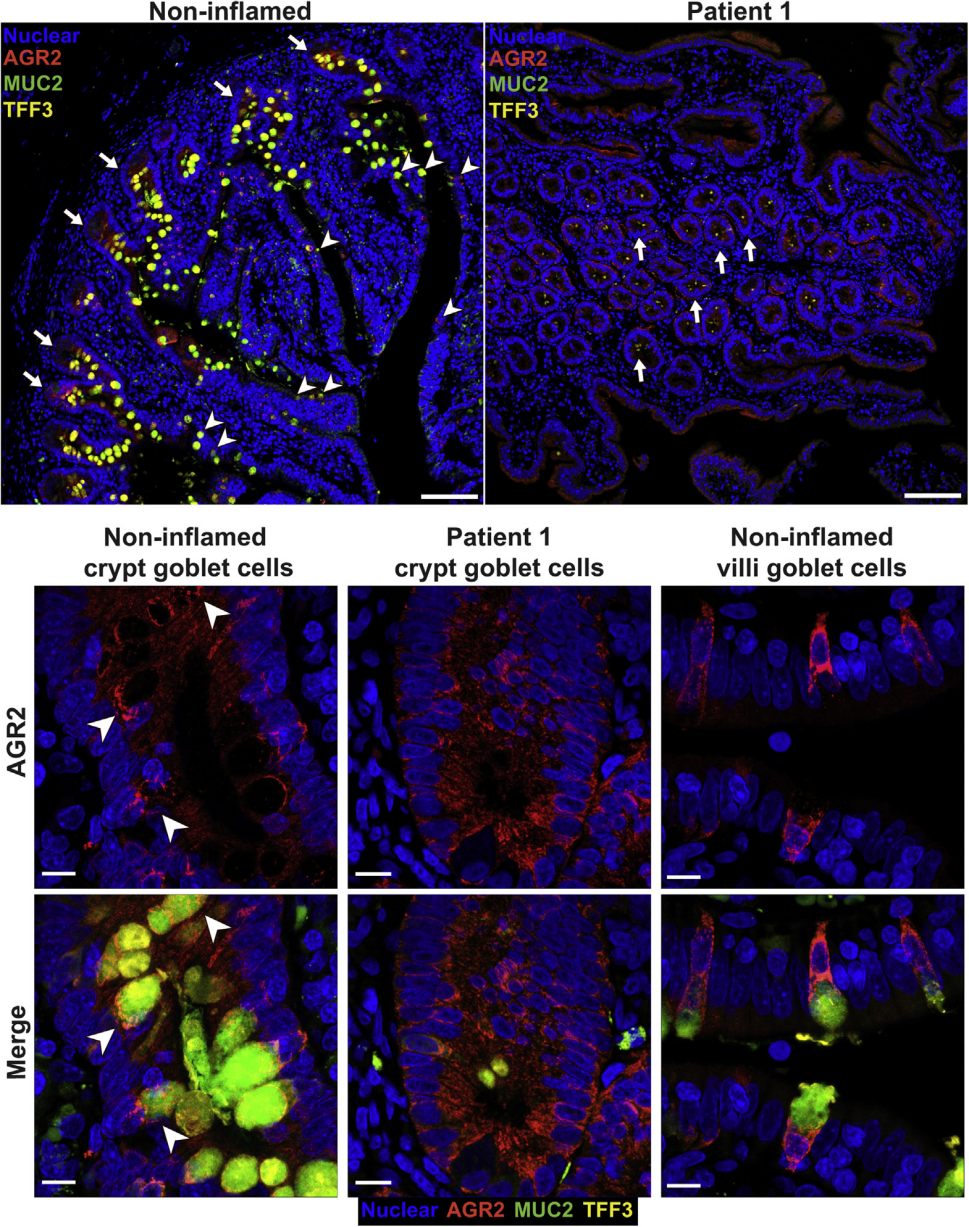
**AGR2 localization changes in the small bowel of patients to encompass all epithelial surfaces.** Fluorescent immunohistochemistry on formalin-fixed paraffin-embedded sections showing AGR2 (red, clone D9V2F) localization and goblet cells, identified by MUC2 (green, clone F-2) and TFF3 (yellow, clone B-1) staining. Nuclei were counterstained with Hoechst 33342 (blue). Images were acquired via confocal microscopy. The 2 large panels are low-magnification images (10× objective) of the small bowel from a noninflamed control and patient 1 and show the change in localization of AGR2 in the patients. In the control, crypts (*arrows*) show a diffuse staining of AGR2 across all cell types, while villi show near-exclusive staining within goblet cells (*arrowheads*). The patient on the other hand shows staining across all epithelial surfaces in the section. Smaller panels below show maximum intensity projection of the high-magnification (63× objective) confocal Z-stacks of representative goblet cells from the noninflamed control and patient 1. For each pair of images, *top panels* show only nuclear (blue) and AGR2 (red) staining, while the *bottom panels* show the merge for all 4 markers. Patient AGR2 staining is diffuse within the cytoplasm and is present throughout all epithelial cells, in contrast to the goblet cell–specific staining in the control. Accumulation of AGR2 against the mucin granules (*arrowhead*) observable in the control is lost in the patient. Identical thresholding parameters were applied for all images. *Scale bars*: 100 μm (large panels); 10 μm (small panels).

**Figure 9:**
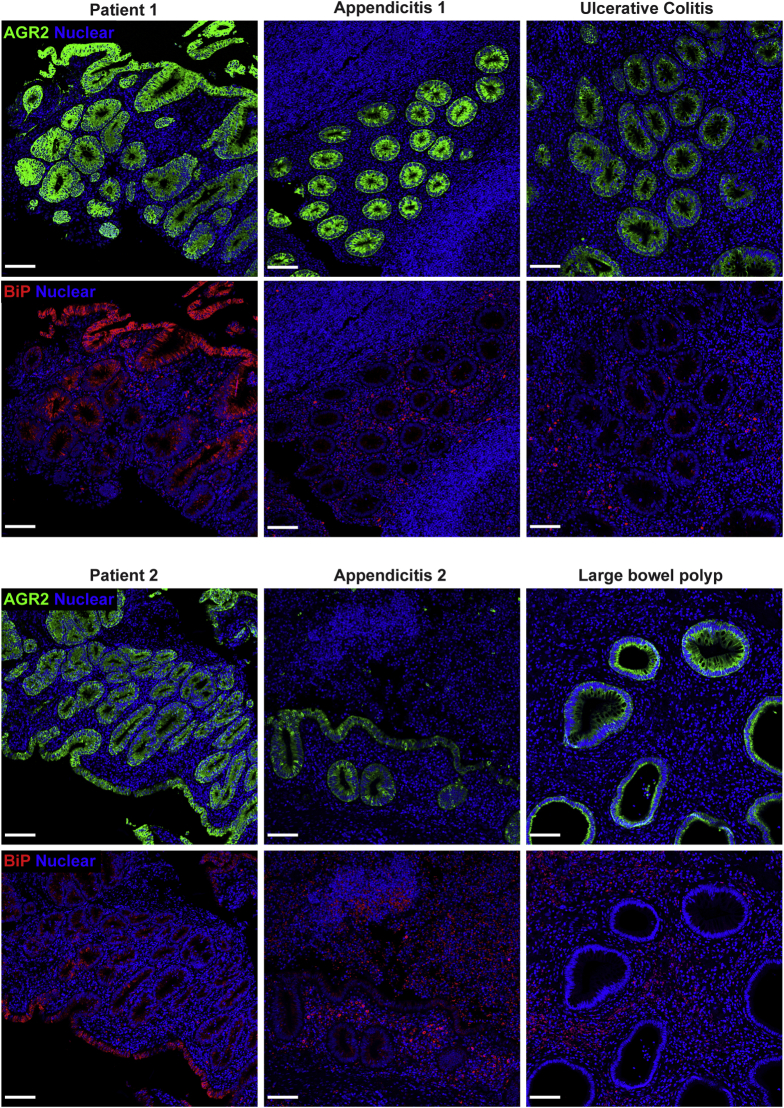
**AGR2 up-regulation in the patients is similar to other inflamed conditions.** Although AGR2 up-regulation occurs in multiple inflammatory conditions, patients 1 and 2 showed a strikingly greater up-regulation of BiP than the other conditions. Large-bowel sections from patients 1 and 2 and patients suffering from appendicitis, polyp, and ulcerative colitis were co-stained for AGR2 (green, clone 5G1.1) and BiP (red, C50B12). Nuclei were stained with Hoechst (blue). Confocal Z stacks were acquired with a 10× objective and visualized using maximum intensity projection. *Scale bars*: 100 μm. When comparing other conditions with the AGR2 mutant patients, we found increased AGR2 to be common in inflammatory conditions of the gut, but the BiP increase was greatest in AGR2 mutant patients. These observations support the impaired capacity of AGR2 H117Y to regulate ER stress.

Staining for AGR2 was performed to assess expression in the patients and to determine if the mutation impacted protein levels. AGR2 protein expression in gastric sections in the patients appeared higher compared with inflamed and noninflamed controls. However, AGR2 localization was altered in patients 1 and 2 because intense staining was localized to all epithelial surfaces, whereas AGR2 staining in the noninflamed and inflamed controls was mainly within the glands (Figure 7). In the small bowel, AGR2 stained more intensely in patients 1 and 2 compared with controls (Figure 7). In noninflamed controls, AGR2 was localized predominantly in goblet cells, with diffuse staining at the base of crypts (Figures 7 and 8). In patients 1 and 2, however, the localization of AGR2 was altered, showing high expression across all epithelial cells (Figures 7 and 8). Large-bowel AGR2 staining also showed increased intensity in patients compared with noninflamed controls, but was comparable with a number of tested inflamed controls (Figures 7 and 9). Our findings show that AGR2 is highly up-regulated in the 2 patients compared with controls and was localized to all epithelial surfaces in all tissues for which histologic sections were available. These data suggest that the AGR2 variant is expressed. Because AGR2 expression is increased upon ER stress, the up-regulation of AGR2 expression in the patients might be associated with defective function and furthermore indicative of ER stress.^26^

### Loss of Gel-Forming Mucins in Intestinal and Gastric Epithelia

**Figure 10:**
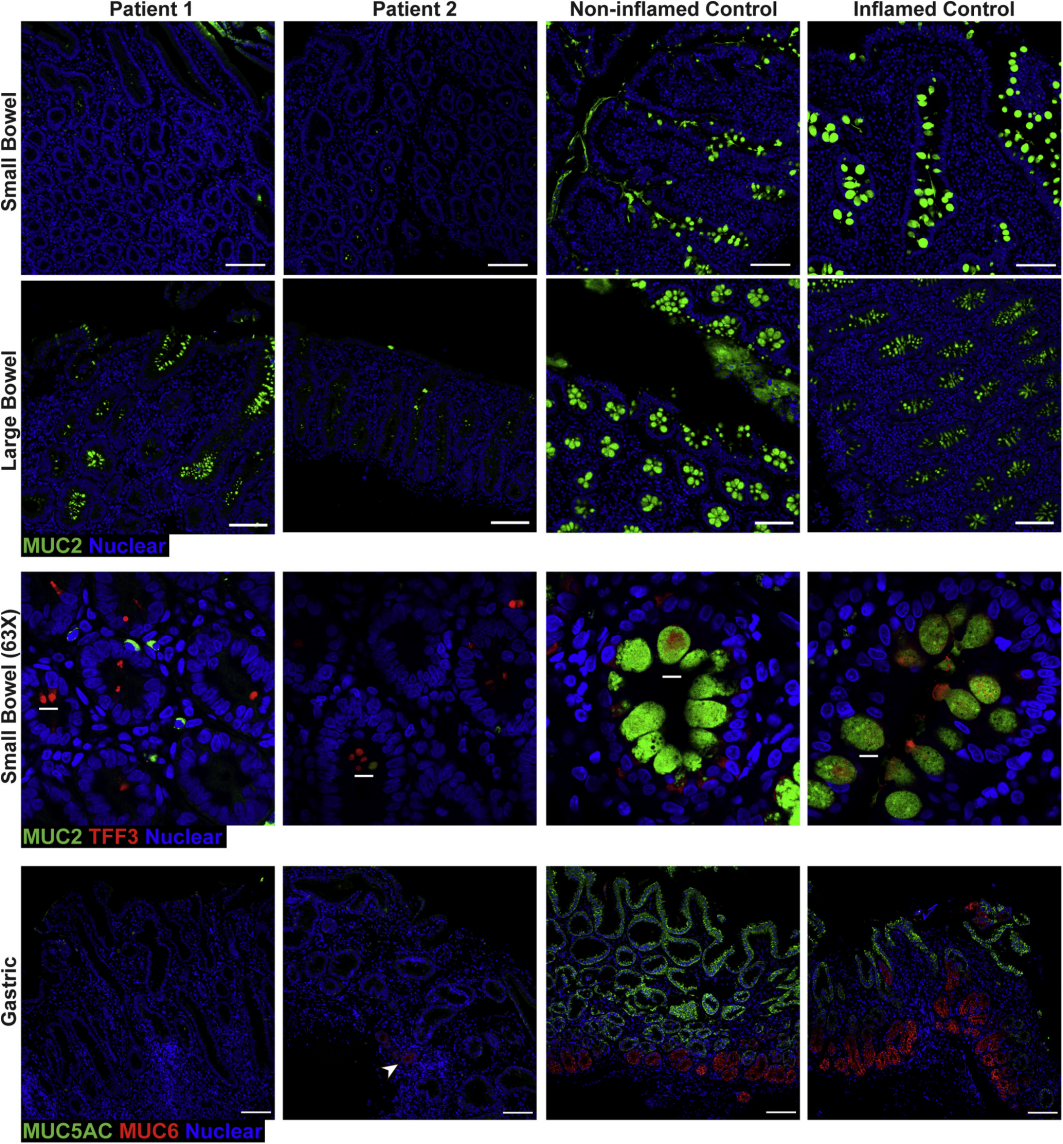
**Depletion of gel-forming mucins MUC2, MUC5AC, and MUC6 in gastrointestinal tissues of the patients.** Fluorescent immunohistochemistry staining of formalin-fixed paraffin-embedded sections showing depletion of MUC2 (green, clone F-2) in small and large bowel, as well as MUC5AC (green, clone CLH2) and MUC6 (red, clone CLH5) in the stomach. Nuclei were stained with Hoechst (blue). Patient 2 shows a small group of cells positive for MUC6 (*arrowhead*). Representative images are shown for patients 1 and 2, 1 of 3 noninflamed controls, and 1 of 5 inflamed controls for small bowel. For large bowel, representative images are shown for 1 of 3 noninflamed controls and 1 of 6 inflamed controls. Representative, high-magnification (63× objective) images of small-bowel crypts show the reduced size of goblet cell thecae in the patients with staining for TFF3 (red, clone B-1) and MUC2 (green, clone F-2). All other images were acquired with a 10× objective. For gastric sections, MUC5AC staining was replicated across 3 noninflamed controls and 4 inflamed controls, while MUC6 staining was replicated across 2 noninflamed controls and 2 inflamed controls. Identical thresholding parameters were applied to the images from each respective tissue. Inflamed conditions shown in the figure are celiac for small bowel, appendicitis for large bowel, and *H pylori* for gastric. Staining for every marker was replicated a minimum of 3 independent times. *Scale bars*: 100 μm (low magnification); 10 μm (high magnification).

AGR2 is known to play a crucial role in the processing of gel-forming mucins.^14^^,^^16^^,^^17^ MUC2 is the predominant gel-forming mucin in the bowels, whereas MUC5AC and MUC6 are major gel-forming mucins in the stomach.^27^ We examined the presence of MUC2 in the bowel and MUC5AC and MUC6 in the stomach to evaluate the impact of AGR2 H117Y on the expression of gel-forming mucins. Staining showed a near-complete loss of MUC2 staining within goblet cell thecae in the small bowel and a substantial reduction in the large bowel of patients 1 and 2 compared with inflamed and noninflamed controls (Figure 10). Examination of remaining goblet cell thecae at high magnification shows a significant reduction in the size of the theca in the patients compared with both inflamed and noninflamed controls (Figure 10). On closer inspection, patient goblet cells appear to even have reduced accumulation of the nonglycosylated MUC2 precursor compared with other inflamed controls both in the small and large bowel (Figures 3*B* and 5). Similarly, we observed a reduction of gastric MUC5AC and MUC6 staining (Figure 10) in the siblings compared with noninflamed and inflamed controls, although patient 2 showed some residual patches of MUC5AC and MUC6 staining. These findings resemble those of the *Agr2* knockout mice, except for the lack of nonprocessed MUC2 accumulated in the goblet cells of the patients.^14^^,^^15^^,^^17^

### Increased Marker of ER Stress in Intestinal and Gastric Mucosa of AGR2 Patients

**Figure 11:**
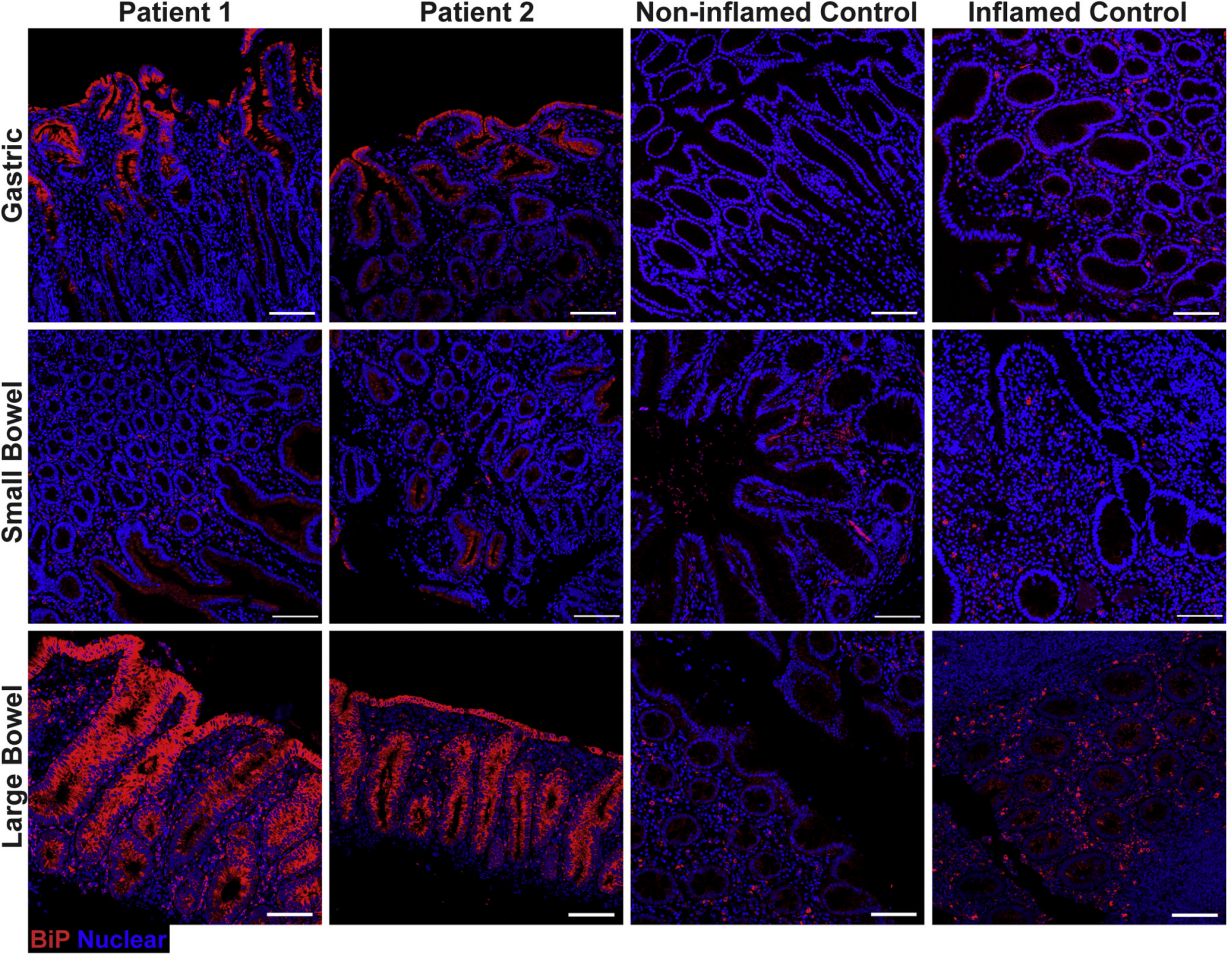
**Increased ER stress in the gastrointestinal mucosa of the AGR2 variant patients.** BiP (red, clone C50B12) abundance was assessed in gastric and intestinal formalin-fixed paraffin-embedded sections by fluorescent immunohistochemistry and confocal microscopy acquired with a 10× objective. Primary antibody was nonconjugated and used with a goat anti-rabbit AF647 secondary antibody. Nuclei were stained with Hoechst (blue). Inflamed conditions are Crohn’s for gastric, Crohn’s for small bowel, and appendicitis for large bowel. *Scale bars*: 100 μm. Representative images from 1 of at least 3 independent experiments are shown for patients 1 and 2. For small bowel, representative images of 1 of 3 noninflamed controls and 1 of 4 inflamed controls were shown. For large bowel, representative images are shown for 1 noninflamed control and 1 of 5 inflamed controls. For gastric sections, representative images are shown for 1 of 3 noninflamed controls and 1 of 4 inflamed controls. Identical thresholding parameters were applied to the images from each respective tissue.

ER stress has been shown to be regulated by AGR2^13^ and induced by misfolding of mucins.^21^ Therefore, we evaluated the ER stress in the gut of patients 1 and 2 by staining for Binding Immunoglobulin Protein (BiP/GRP78/HSPA5), a marker of ER stress, via fluorescent immunohistochemistry (Figure 11). In the patients, increased BiP expression was observed in the epithelial cells of the small bowel, large bowel, and gastric sections compared with inflamed and noninflamed controls (Figure 11). BiP staining was brightest at the luminal surfaces of all 3 tissues from the patients compared with both noninflamed and inflamed controls (Figure 11). These results suggest that despite having similarly increased levels of AGR2 compared with inflammatory controls, there was a greater degree of ER stress in the 2 siblings.

### AGR2 p.H117Y Reduces Binding to MUC2 and Impairs the ER Stress Response In Vitro

**Figure 12:**
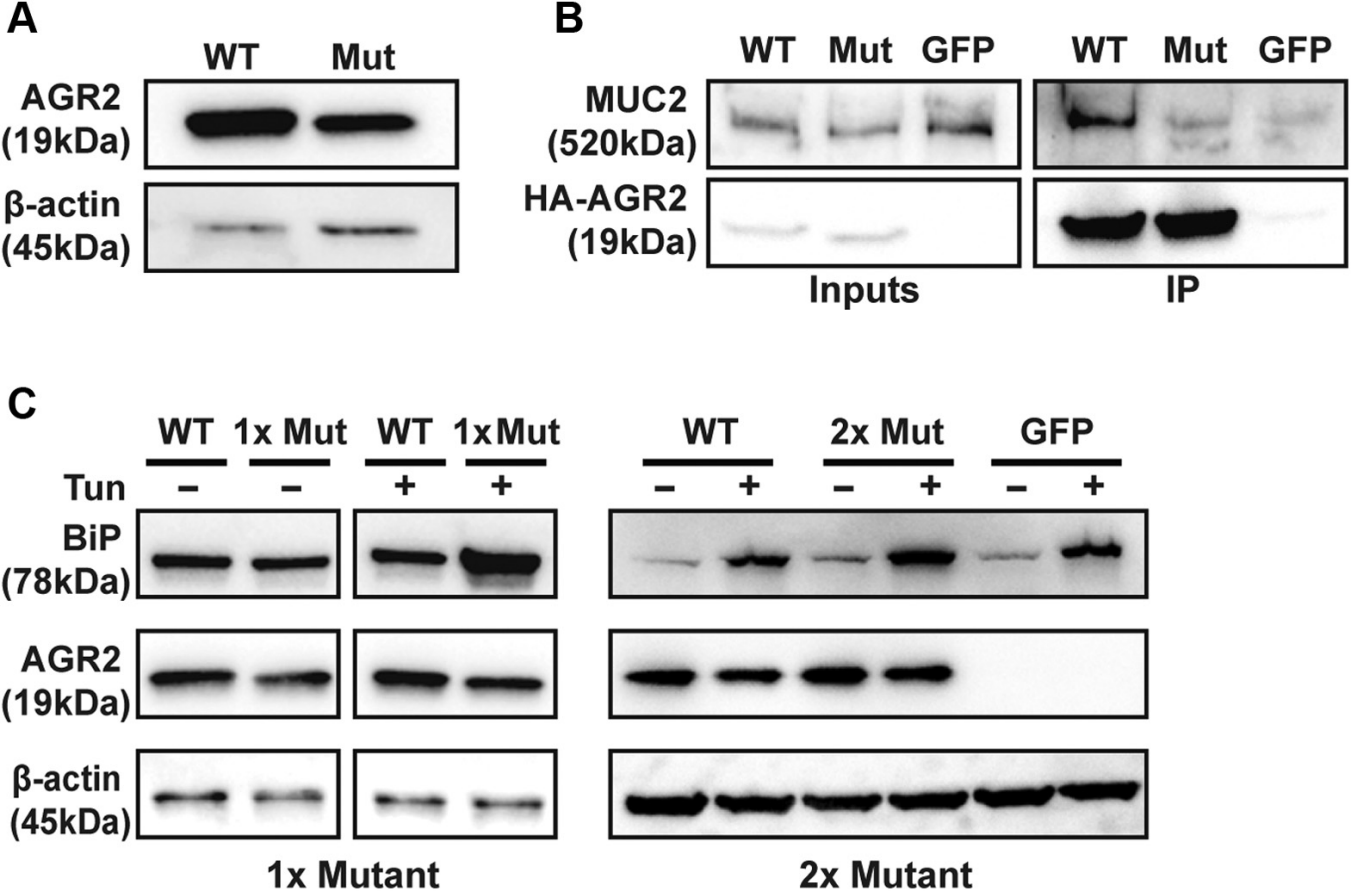
**AGR2 H117Y has a reduced ability to interact with MUC2 and regulate ER stress in vitro.** (*A*) HEK293T cells were transfected with equal amounts of WT or H117Y mutant (Mut) HA-tagged AGR2 (HA-AGR2) and blotted for AGR2 or β-actin as a loading control. Mutant HA-AGR2 consistently showed lower expression when examined by Western blot. Data are representative of 3 independent experiments. (*B* and *right panel* of *C*) Mutant AGR2 was transfected at double the amount compared with WT plasmid to obtain the same level, or higher, of mutant AGR2 protein as WT. (*B*) LS174T cells were transfected with WT or H117Y mutant HA-AGR2 or Green Fluorescent Protein (GFP) as a transfection control. Transfected AGR2 was immunoprecipitated using anti-HA beads, and immunoprecipitates were blotted for MUC2 (clone CCP58) and HA-tag. (*C*) Immunoblots of the lysates of HEK293T cells overexpressing WT or Mut AGR2 or GFP and stressed with tunicamycin (Tun) 48 hours after transfection. *Left*: Data from cells transfected with equal amounts of WT or Mut (1×) plasmid. *Right*: Results in which twice (2×) the amount of mutant AGR2 plasmid was transfected than for WT. Blots were stained with antibodies against BiP (clone C50B12), AGR2 (clone D9V2F), HA (clone C29F4), and β-actin. Experiment was repeated 3 independent times in HEK293T.

The loss of mucins and increased ER stress in the gastrointestinal tract of patients 1 and 2 suggest that AGR2 variant p.H117Y may disrupt processing of MUC2 and impair ER stress regulation. To evaluate the function of AGR2, we used hemagglutinin (HA)-tagged overexpression constructs of wild-type (WT) and H117Y mutant AGR2. Mutant AGR2 levels were consistently lower than WT on Western blot (Figure 12*A*). To examine the function of mutant AGR2 independent of the reduced expression, the amount of mutant AGR2 plasmid was doubled in subsequent transfections compared with WT plasmid to yield equal, or higher, levels of mutant AGR2 protein (Figure 12*B* and *C*).

To investigate function, we first examined the binding of AGR2 H117Y to MUC2 in LS174T cells, a goblet-like cell line that constitutively expresses high levels of MUC2. When overexpressing HA-tagged, WT AGR2 or the H117Y mutant in LS174T cells, less MUC2 co-immunoprecipitated with the H117Y mutant than WT AGR2 (Figure 12*B*). Next, the impact of the AGR2 H117Y mutant on ER stress regulation was tested. To evaluate the ER stress regulatory function of mutant AGR2 independently from its role in mucin processing, we used Human Embryonic Kidney 293T (HEK293T) cells, which do not express gel-forming mucins or endogenous AGR2. WT AGR2 or the H117Y mutant were overexpressed in HEK293T cells, and ER stress was induced by challenging the cells with tunicamycin (4 mg/mL) for 6 hours. ER stress levels were assessed by evaluating BiP abundance via Western blot (Figure 12*C*). Cells overexpressing WT AGR2 showed lower levels of BiP compared with those expressing the H117Y mutant AGR2.

In summary, these findings indicate that *AGR2* p.H117Y shows reduced expression, indicating that the increased expression seen in vivo is owing to enforced expression. It furthermore suggests that the p.H117Y variant has reduced capacity to bind MUC2 and also is less capable of regulating ER stress compared with WT AGR2, independent of its role in gel-forming mucin processing.

## Discussion

In this study, we report on a biallelic AGR2 variant that causes mucus barrier impairment, congenital diarrhea, and infantile IBD. The enteropathy was associated with an extensive depletion of goblet cells indicated not only by H&E but also by the more sensitive staining of TFF3, CLCA1, and processed and unprocessed MUC2. The ability of goblet cells to differentiate in the patients was evident by finding small numbers of goblet cells in the small bowel and more abundant goblet cells in the large bowel. However, the diminished count still could be attributed to the depletion of the markers we used to detect the goblet cells. The striking resemblance of the patient phenotype and the *Agr2* knockout mouse model (summarized in Table 4) alongside the functional data we describe herein suggest that the missense mutation in AGR2 is disease-causative owing to its loss of function.^14^^,^^15^^,^^17^ These similarities to the knockout model are striking despite the increased expression of AGR2 in the patients. Inflammation is known to increase the expression of AGR2^16^ and augment the production of mucus, which is defective in the patients. Thus, the mucus barrier impairment and ensuing inflammation is the likely driver of AGR2 up-regulation in the patients.

**Table 4:** Comparison Between AGR2 KO Mice and Patient Phenotype

Location	Phenotype	KO mouse	Patients
Clinical	Diarrhea onset	Not reported	Congenital
Stomach	General phenotype	Hyperplastic^17^	Severe intestinal metaplasia
	Paneth cells	Not reported^17^	Present (metaplastic)
	Parietal cells	Lost^17^	Lost
	MUC6	Not reported^17^	Severely diminished
	ER stress	Not significant^a^^,^^17^	Up-regulated^b^
Small bowel	Paneth cells	Hyperplastic^14^	Normal
	Goblet cells	Severely diminished^14^^,^^15^	Severely diminished
	Glycosylated MUC2	Severely diminished^14^^,^^15^	Severely diminished
	ER stress	Up-regulated^a^^,^^14^	Up-regulated^b^
Large bowel	Colitis	Severe^14^	Severe
	Goblet cells	Diminished^14^^,^^15^^,^^22^	Diminished^c^
	Glycosylated MUC2	Severely diminished^14^^,^^15^^,^^22^	Severely diminished
	ER stress	Not reported	Highly up-regulated^b^

KO, knockout.

a Based on RNA expression.

b Based on BiP by immunohistochemistry.

c Patient intervariability in severity.

Goblet cells are one of 2 secretory cell types that have been shown to be particularly susceptible to ER stress as seen in the *Xbp1* knockout mouse model.^20^ Moreover, goblet cells are responsible for processing large quantities of MUC2 ahead of secretion. The mouse models *Winnie* and *Eeyore* show the demand placed by MUC2 processing on the ER of goblet cells because the MUC2 missense mutations in these mice result in MUC2 misfolding and increased levels of goblet cell–specific ER stress and apoptosis.^21^ In the intestinal mucosa, AGR2 is localized primarily to goblet cells and aids in the processing of MUC2, therefore assisting in the maintenance of goblet cell ER homeostasis. *Agr2* knockout mice show a depletion of goblet cells without depleting Paneth cells.^15^ Deficiency in AGR2 would make goblet cells particularly susceptible to ER stress and this is exacerbated in an inflamed state because ER stress increases during inflammation.^28^ It is crucial to clarify, however, that the mutation in AGR2 likely does not cause ER stress directly. Instead, it appears to be defective in reducing ER stress when combined with an insult(s). This is evident in our in vitro model in which overexpressing AGR2 H117Y does not appear to increase ER stress significantly compared with the WT until challenged with tunicamycin.

In our patients, we also observed the loss of 2 major mucin-producing cell types in the stomach. Foveolar cells and mucous neck cells are responsible for producing MUC5AC and MUC6, respectively, and both cell types were not observable on H&E stains and staining for their respective mucins was greatly diminished. In addition, parietal cells and their marker H^+^/K^+^ ATPase also were missing, which is consistent with the H&E staining and the findings in the knockout mouse model.^17^ Instead, the patients presented with severe intestinal metaplasia containing Paneth cells in the gastric mucosa. Metaplastic Paneth cells are not a common feature of intestinal metaplasia.^29^ Considering our patients developed this severe metaplasia at as early as 2 months of age, this feature highlights the overall unique phenotype of these patients.

Defective processing and loss of MUC2 lead to defective mucus barrier integrity and inflammation.^6^^,^^7^ The substantial depletion of MUC2 observed in the epithelia of our patients suggests a mucus barrier defect with consequences for bacterial translocation that drives subsequent intestinal inflammation. However, we were unable to observe this mucus barrier defect based on available routinely processed histology sections because the secreted mucins that form the mucus barrier are dissolved during the formalin-fixation step of tissue processing.

The increased epithelial BiP levels in our patients suggest a deficiency in ER stress regulation, indicating that the loss of AGR2 function cannot be compensated for by the up-regulated expression. In line with this hypothesis is our finding that AGR2 H117Y has a reduced capacity to regulate ER stress even in the absence of gel-forming mucins in vitro. Impaired binding of AGR2 H117Y to MUC2 also was evident, which indicates a defect in mucin processing in the patients that, in turn, would contribute to increased ER stress. We posit that AGR2 loss of function in our patients results in increased ER stress driven by the luminal insults resulting from the mucus barrier defect. The AGR2 defect and heightened ER stress affects goblet cells most severely, leading to their selective depletion by apoptosis.

In summary, we describe a monogenic **E**nteropathy caused by **A**GR2 deficiency, **G**oblet cell **L**oss and **E**R **S**tress (EAGLES). The 2 patients show a unique disease phenotype characterized by a striking depletion of goblet cells; loss of gel-forming mucins MUC2, MUC5AC, and MUC6; heightened ER stress in the gastrointestinal tract; and severe intestinal metaplasia of the gastric epithelium. The AGR2 variant of the patients results in a loss of function. The combined evidence in human beings and animal models suggests that AGR2 deficiency causes a disruption to gel-forming mucin processing and subsequent exacerbation of ER stress in mucin-producing cells, such as goblet cells. Our findings highlight the crucial role of AGR2 in preserving goblet cell health and function as well as the importance of the mucus barrier in maintaining gut homeostasis.

## Materials and Methods

### Patients and Specimens

The study protocol for the collection of patient specimens and data was approved by the Sidra Medicine Institutional Review Board. Written informed consent was obtained from the patient’s family before data or specimens were collected.

Blood from the parents and the patients was collected into Vacutainer ACD Tubes (Becton Dickinson, Franklin Lakes, NJ). Peripheral blood mononuclear cells were separated from whole blood using Ficoll-Paque PLUS (GE Healthcare, Uppsala, Sweden) gradient. Buccal swab samples from healthy siblings were collected using ORAcollect for Pediatrics (DNA Genotek, Ottawa, Canada). Histology samples were obtained from biopsy paraffin blocks retrieved for clinical purposes at the age of 1 year and age 2 months for patient 1 and patient 2, respectively. For immunohistochemistry, pediatric noninflamed and inflamed control samples for gastric (including IBD, n = 2; *Helicobacter pylori* gastritis, n = 2), small bowel (including IBD, n = 2; focal duodenal inflammation, n = 1; Familial Mediterranean fever, n = 1; duodenitis, n = 1; and celiac disease, n = 1), and large bowel (including IBD, n = 1; appendicitis, n = 3; celiac, n = 1; colitis, n = 1; and polyp, n = 1) were obtained from surplus specimens originally collected for diagnostic purposes or during surgical procedures. For goblet cell quantification, scans of diagnostic H&E sections of patients younger than age 10 years were used. All control samples were deidentified before being provided for research purposes according to institutional policy. Noninflamed and inflamed sections were chosen on the basis of histologic morphology and clinical history.

### DNA Extraction, Sequencing, and Variant Identification

DNA was extracted from peripheral blood mononuclear cells using the DNeasy Blood and Tissue Kit (Qiagen, Hilden, Germany). Genomic DNA was extracted from buccal swab samples using PrepIT-L2P (DNA Genotek) according to the manufacturer’s recommendations. DNA from both parents and affected siblings were whole-genome sequenced to an average coverage of 30 reads per base on the Illumina (San Diego, CA) HiSeq X system in the Genomics core facility at Sidra Medicine. Reads were aligned to the reference human genome version hg19 using the Burrows-Wheeler Aligner. Single-nucleotide variant and indel calling were performed using the Genome Analysis Toolkit (GATK) HaplotypeCaller. For variant quality filtering, GATK Variant Quality Score Recalibration was used. SnpEff/SnpSift was used to annotate the Variant Call Format (VCF) files. Only nonsynonymous, nonsense, indel, and splicing variants were considered. Filtering was based on variant quality, multiple models of inheritance, allele frequency (<0.005; 1000 Genomes Project, The Genome Aggregation Database), and predicted deleteriousness (SIFT, PolyPhen, MutationTaster (Charité, Berlin, Germany)) to prioritize high-quality missense and likely pathogenic variants. Variants were lastly prioritized based on clinical correlation. For Sanger sequencing, genomic DNA samples were used as template for polymerase chain reaction and amplification was performed using the following primers:

forward: 5’-CCATTCAGCACTTTATCTCATGTTCTGC-3’; reverse: 5’-CGAGGCCTCTCGTATTCAGG -3’. Polymerase chain reaction products were sequenced using the Big Dye Terminator v3.1 Kit (Applied Biosystems, Waltham, MA). Chromatograms were analyzed using the open-source software Unipro UGENE v36 (http://ugene.net/download-all.html).

### Structural Modeling of the H117Y Mutant of AGR2

Structural modeling was performed using the structure of WT human AGR2 (residues 41–175; Protein Data Bank accession number: 2LNS) solved by Patel et al^30^ as the template. The H117Y variant was introduced using the ICM software package (Molsoft, San Diego, CA).

### Quantification of Goblet-to-Total Epithelial Cell Ratio

Deidentified diagnostic scans of H&E-stained sections from pediatric patients younger than age 10 years were obtained. Small-bowel controls included noninflamed (n = 8) and inflamed controls consisting of IBD (n = 2) and celiac disease (n = 1). Large-bowel controls included noninflamed (n = 3), and IBD (n = 8) as inflamed control. Three random regions from each scan were selected using the open-source software QuPath 0.2.0-m4 (https://qupath.github.io/). Scans were blinded and then all columnar epithelial and goblets cells within the selected regions were counted and annotated manually using QuPath 0.2.0-m4. Columnar cell nuclei and goblet cell thecae were counted. The ratio was calculated as the total number of goblet cell thecae from all 3 regions in each slide divided by the total number of columnar epithelial cell nuclei from the same regions. We counted a minimum of 419 epithelial cells per slide.

### Immunohistochemistry and Confocal Microscopy

Biopsy samples were fixed in 10% neutral-buffered formalin for a maximum of 24 hours and then processed and embedded in paraffin blocks. Sections from formalin-fixed paraffin-embedded blocks were dewaxed using xylene, then rinsed with 100% ethanol, and rehydrated with 95% ethanol and then 100% double-distilled water. Retrieval of antigens was performed by microwave heating in Antigen Unmasking Solution, Citric Acid Based (Vector Laboratories, Burlingame, CA), followed by permeabilization using 0.1% Triton X-100 (VWR, Radnor, PA) in Tris-buffered saline (TBS). To stain, sections first were blocked with 5% fetal calf serum and 1% bovine serum albumin in TBS for 1 hour at room temperature, and then incubated overnight at 4ºC with unconjugated primary antibodies followed by incubating with fluorophore-conjugated secondary antibodies for 1 hour at room temperature. A second blocking step was performed to saturate free binding sites of the secondary antibodies by incubating in 5% normal mouse serum (Sigma, St. Louis, MO) plus 1% bovine serum albumin in TBS for 1 hour at room temperature. Sections then were stained with fluorophore-conjugated primary antibodies and Hoechst 33342 (Thermo Fisher Scientific, Waltham, MA) overnight at 4ºC. All stained sections were acquired using the LSM 780 (Zeiss, Jena, Germany). Replicates were performed by staining the patients alongside a minimum of 1 noninflamed and 1 inflamed control in each replicate. Choice of fluorophore for both primary and secondary antibodies varied depending on the panel in which each respective marker was replicated, but was kept the same across all samples within each respective replicate experiment. The specific antibodies used for the images included in the figures are detailed in the respective figure legend. The number of noninflamed and inflamed controls and their respective conditions varied between replicates depending on human tissue availability. Thresholding, scale bar generation, maximum intensity projection, and image exporting were performed in Zen Blue (Zeiss). A complete list of antibodies can be found in Table 5.

**Table 5:** List of All Antibodies Used in This Study

Antibody name	Company	Product number	Application
Anti-rabbit IgG (H+L) antibody, human serum adsorbed and peroxidase-labeled	Seracare (Milford, MA)	5450-0010	WB
Anti-mouse IgG (H+L) antibody, human serum adsorbed and peroxidase-labeled	Seracare (Milford, MA)	5450-0011	WB
Goat anti-mouse IgG (H+L) cross-adsorbed secondary antibody, Alexa Fluor 546	Thermo (Waltham, MA)	A-11003	IHC
Goat anti-rabbit IgG (H+L) highly cross-adsorbed secondary antibody, Alexa Fluor Plus	Thermo (Waltham, MA)	A32733	IHC
Lysozyme Polyclonal Antibody	Fisher Scientific (Waltham, MA)	PA516668	IHC
Mucin 5AC antibody (CLH2) Alexa Fluor 594	Santa Cruz (Dallas, Texas)	sc-33667 AF594	IHC, WB
HRP-conjugated β actin monoclonal antibody	Proteintech (Rosemont, IL)	HRP-60008	WB
BiP (C50B12) rabbit mAb	Cell Signaling (Danvers, MA)	3177S	IHC, WB
ITF antibody (B-1) Alexa Fluor 594	Santa Cruz (Dallas, TX)	sc-398651 AF594	IHC
Mucin 5AC antibody (CLH2)	Santa Cruz (Dallas, TX)	sc-33667	IHC
Mucin 2 antibody (F-2)	Santa Cruz (Dallas, TX)	sc-515032	IHC
AGR2 (6C5)	Santa Cruz (Dallas, TX)	sc-101211	WB
AGR2 (D9V2F) XP rabbit mAb	Cell Signaling (Danvers, MA)	13062	IHC, WB
Anti-AGR2 antibody, clone 5G1.1	Millipore (Burlington, MA)	MABT122	IHC
HA-Tag (C29F4) rabbit mAb	Cell Signaling (Danvers, MA)	3724S	IP, WB
Anti-mucin 6/MUC6 antibody (CLH5) Alexa Fluor 546	Santa Cruz (Dallas, TX)	sc-33668 AF546	IHC
CLCA1 (EPR12254-88)	Abcam (Cambridge, United Kingdom)	ab180851	IHC
H+/K+ ATPase β antibody (C-4) Alexa Fluor 546	Santa Cruz (Dallas, TX)	sc-374094 AF546	IHC
MUC2 antibody (CCP58)	Novus (Littleton, CO)	NBP2-25221	IP, WB

ATPase, adenosine triphosphatase; H+L, Heavy and Light chain; HRP, horseradish peroxidase; IHC, immunohistochemistry; IP, immunoprecipitation; mAb, monoclonal antibody; WB, Western blot.

### Site-Directed Mutagenesis and Sequencing

For AGR2 overexpression, pPM-C-HA-AGR2 (BC015503) (Applied Biological Materials, Richmond, British Columbia, Canada) was used. To introduce the H117Y mutation, the QuikChange Lightning Site-Directed Mutagenesis Kit (Agilent, Santa Clara, CA) was used according to the manufacturer’s protocol using the following primers: 5’-CATCAGGAGAAAGGTATTTGTCAGTTGTTTCATAAACCAGATTGA-3’ and 5’-TCAATCTGGTTTATGAAACAACTGACAAATACCTTTCTCCTGATG-3’. Sanger sequencing was performed to confirm successful mutagenesis using the following primers: 5’-GGGGTGACCAACTCATCTGG-3’, 5’-AACATAATCCTGGGGACATACTGG-3’, 5’-CGCAAATGGGCGGTAGGCGTG-3’, 5’-TCTCCTGATGGCCAGTATGTC-3’, and 5’-CGGTTTGAATATCTTCCAGTGA-3’.

### Immunoprecipitation and Western Blot

LS174T cells were split and cultured to subconfluence in Opti-MEM media (Thermo Fisher Scientific). Twenty-four hours later, cells were transfected with plasmids expressing HA-tagged WT AGR2 (12 mg for 10-cm plate), AGR2 H117Y (24 mg for 10-cm plate), or green fluorescent protein (as a control for transfection efficiency) using Lipofectamine LTX (Invitrogen, Waltham, MA) in Opti-MEM media; 4 hours later media was changed to RPMI media containing 10% fetal bovine serum. Forty-eight hours after transfection, cells were harvested and lysed in lysis buffer containing (10 mmol/L HEPES, 150 mmol/L NaCl, 1 mmol/L ethylene glycol-bis(β-aminoethyl ether)-*N,N,N′,N′*-tetraacetic acid, 0.1 mmol/L MgCl_2_, 0.5% Triton X-100, pH 7.4). For immunoprecipitation of HA-tagged AGR2 proteins, 2.5 mg total cell lysate was incubated with anti-HA magnetic beads (Pierce, Waltham, MA) for 2 hours at 4ºC. Beads then were washed 4 times with immunoprecipitation wash buffer (1× lysis buffer + 0.1% Triton X-100) and then boiled at 90ºC for 5 minutes in 1× Laemmlli buffer. Eluates then were loaded onto 4%–20% TGX gels (Bio-Rad, Hercules, CA) alongside 50 mg lysate as input for each condition. Proteins then were transferred onto nitrocellulose membrane and blotted with antibodies against proteins of interest. Table 5 lists all antibodies used.

### Cell Culture and ER Stress

HEK293T cells were cultured to subconfluence in Advanced RPMI media (Thermo Fisher Scientific, Waltham, MA), in 24-well plates (Corning, Corning, NY). Cells then were transfected with overexpression constructs expressing WT and mutant AGR2 (described earlier) using Turbofect (Thermo Fisher Scientific, Waltham, MA). At 24–48 hours after transfection the cells were treated with 4 mg/mL tunicamycin (Cayman Chemical, Ann Arbor, MI) or dimethyl sulfoxide (MilliporeSigma, St. Louis, MO) for 6 hours before being harvested for Western blot.

### Graphs and Statistical Analysis

Graph generation and CI calculations were performed using GraphPad Prism 8 (GraphPad Software, San Diego, CA).
