# Supplementary material for: Human AGR2 Deficiency Causes Mucus Barrier Dysfunction and Infantile Inflammatory Bowel Disease
Source: Cell Mol Gastroenterol Hepatol. 2021 Jul 6;12(5):1809–30. doi: 10.1016/j.jcmgh.2021.07.001 (PMC8551217; doi:10.1016/j.jcmgh.2021.07.001)
Supplement: Supplementary Methods [file mmc1.docx]

Supplementary Materials

Full Case report

Patient 1:

A 5 years old boy, who was born at term by normal vaginal delivery from a first-degree consanguineous parent. His birth weight was 3.3kg. He was breast fed exclusively for the first 6 weeks before cow milk-based formula was introduced. His parents noticed diarrhea since birth with a frequency of up to 4 times a day. However, upon formula introduction at 6 weeks, his diarrhea got worse and went up to 6 times a day without associated mucus or blood in the stool. He failed to thrive until he was admitted to hospital at the age of 3 months with increasing diarrhea and vomiting and associated dehydration. He was found to be marasmic weighing 4.38 kg at the age of 3 months without obvious dysmorphism. A trial on extensively hydrolyzed formula was not successful; on amino acid-based formula, his diarrhea slightly improved but he failed to gain weight. At the time of his presentation, he had 5 siblings, boys and girls, who were all normal and there was no known history of early childhood deaths and no family history of similar presentations.

He underwent upper and lower GI endoscopy that revealed upper and lower GI mixed chronic and active cellularity inflammation with clear goblet cell depletion and apoptosis. He was therefore started on parenteral nutrition and intravenous methyl prednisolone and azathioprine. There was slight improvement and slow weight gain.

During his admission he was noted to be tachypneic without evidence of acute respiratory infection. A CT scan of his chest revealed bilateral upper chest infiltration with left lower atelectasis. A plan for lung biopsy was declined by his parents. His sweat test was normal, and extensive immune work up revealed no evidence of cellular or humoral immune deficiency. His autoimmune profile was negative. However, he had multiple line infections that were difficult to eradicate. He received parenteral nutrition and NG feeding for 10 weeks, before the parents decided to travel back home. He went home on NG feeds and a tapering dose of azathioprine.

He was lost to follow up until he presented a year later with severe failure to thrive and worsening diarrhea, as the parents were unable to afford amino acid-based formula and were not able to give azathioprine. He had another pulse of steroids, restarted azathioprine, and was started on amino acid-based formula as his parents declined parenteral nutrition. A repeat upper and lower GI endoscopy showed similar microscopic changes with more goblet cell depletion and apoptosis.

Unfortunately, at the age of 2 years, he presented to the emergency department with a severe episode of gastroenteritis and had cardiac arrest with associated hypoxic ischemic encephalopathy and ended up as quadriplegic cerebral palsy. He became dependent on BiPAP and had a gastro-jejunal tube inserted and maintained on azathioprine. However, he failed to achieve a good level of 6-TGN despite increasing his azathioprine dose up-to 3.5mg/kg. He continues to need regular bronchodilators and regular suctions of his airways. He had multiple intensive care admissions due to respiratory problems, which made subjecting him for another endoscopy not possible. However, he had an upper and lower GI endoscopy at the age of 5 years which showed near complete absence of goblet cells from both his upper and lower GI endoscopy with persistent inflammation and apoptosis.

Patient 2:

This patient is the younger brother to Patient 1. He is 3 years old now. He was born at term; his weight was 2.64 kg, and he was breast fed initially but was again symptomatic from day one with frequent diarrhea. Because of his brother’s history, he was kept in the hospital and was on exclusive breast feeding before he was switched very early to an amino acid-based formula which helped in improving his symptoms to some extent. Because of poor weight gain, he underwent upper and lower GI endoscopy at the age of 2 months, which showed identical features to his brother with goblet cells depletion, and mixed active and chronic inflammatory cells infiltrate. He was started on steroids and azathioprine with some improvement of his motion but had slow weight gain.

Again, he had full immunology and autoantibody screening, which did not reveal evidence of cellular or humoral immune deficiency. He had multiple viral respiratory infections and was diagnosed to have reactive airway disease and was maintained on nebulizers and bronchodilators. However, he did not have tachypnoea and did not have respiratory tract problems apart from persistent and recurrent wheezing, snuffly nose, and thick nasal secretions.

Because of his poor weight gain, recurrent episodes of diarrhea and vomiting and frequent hospital admissions, he had parenteral nutrition for 8 weeks before a gastrostomy tube was inserted. He had multiple central line infections and gastrostomy site infection and became MRSA positive. His weight gain fluctuated and, again, parents declined long term parenteral nutrition. Like his brother he never achieved normal 6-TGN level despite escalating his dose of azathioprine to 3.5 mg/kg.

Over the last year there has been some improvement of his weight gain and a reduction of his diarrhea. However, his repeated endoscopies at 2 years of age showed worsening inflammatory gut disease with absent goblet cells and ongoing inflammatory changes with apoptosis.
